# Digital teaching competence of higher education professors: self-perception study in an Ecuadorian university

**DOI:** 10.12688/f1000research.139064.1

**Published:** 2023-11-20

**Authors:** Jenniffer Sobeida Moreira-Choez, Jimmy Manuel Zambrano-Acosta, Alexander López-Padrón

**Affiliations:** 1 Postgraduate Institute, Technical University of Manabí, Portoviejo, Manabí, 130101, Ecuador

**Keywords:** Digital competences, university professors, self perception, educational modality, information and Communication Technologies

## Abstract

**Background:**

Teaching professionalization aimed at the digital transformation of educational scenarios and training processes for students in contemporary higher education requires the mastery of digital competence by the teaching staff. The objectives of the study were to analyze the self-perceived level of digital teaching competence (DTC) of the faculty of the Technical University of Manabí (UTM), Ecuador, and to establish the relationship between age, sex, and academic profile variables with digital teaching competence.

**Methods:**

A quantitative methodological approach was adopted to develop a descriptive-correlational field study with a non-experimental design. The participants were 277 professors, selected through non-probabilistic and voluntary sampling, who completed the DigCompEdu Check-In questionnaire sent by e-mail.

**Results:**

The data showed that the integrator and expert categories obtained high levels in all competencies. There is also a difference in the pedagogy variable by the interaction of sex and academic profile.

**Conclusions:**

It is concluded that the competences self-perceived by the professors are within the intermediate categories such as integrator and expert. Likewise, the age, sex, and academic profile variables differ in the digital pedagogy level, which produces an inconsistent relationship, with the exception of the variable evaluates and provides feedback, where it was significant.

## Introduction

Technology plays a crucial role in contemporary and future society, with significant implications for the educational field. ^1^ The rapid pace of technological change has introduced profound challenges in teaching and learning. ^2^ As a result, educators must recognize the necessity of self-directed professional development to enhance their expertise, including achieving an adequate level of digital literacy. This proficiency is essential for success across various dimensions of education and training. ^3^

As key institutions responsible for fostering the development of professional competence, universities must possess qualified resources, both in terms of materials and human capital, to prepare well-rounded professionals. ^4^^–^^6^ These professionals should be equipped with problem-solving skills, critical thinking abilities, and the right attitudinal disposition necessary to navigate the complexities of the digital age. However, despite the acknowledged importance of technology in education, there are significant gaps in research regarding how educators can effectively select and utilize technological tools within their teaching practices.

Therefore, it becomes crucial for educators to develop proficiency in selecting appropriate technological tools and seamlessly integrating them into their instructional approaches. By doing so, they can plan and implement activities that foster learning environments conducive to student success. ^7^ Unfortunately, there is a dearth of comprehensive research that explores how educators can fully leverage the potential of technology to enhance teaching and learning in the classroom.

Hence, this study aims to address these research gaps by examining educators' self-perception of their digital competences and their capacity to effectively select and utilize technological tools in their teaching practice. By gaining a better understanding of the challenges and needs faced by educators in this context, more effective training and support strategies can be developed to foster their professional development and enhance the quality of education in the digital age.

The findings of this study will have significant implications for educational institutions, policymakers, and educators themselves. Understanding educators' self-perception of their digital competences will shed light on their level of confidence and awareness regarding the use of technology in education. Furthermore, identifying the challenges they face in selecting and utilizing technological tools will provide valuable insights into the barriers they encounter in integrating technology effectively into their teaching practice.

Based on these insights, tailored training programs can be developed to address the specific needs of educators, helping them acquire the necessary skills and knowledge to make informed decisions about technology integration. Additionally, the study will contribute to the development of guidelines and best practices for selecting and utilizing technological tools, benefiting both novice and experienced educators in their pedagogical endeavors.

Ultimately, the aim of this study is to improve the educational landscape by equipping educators with the tools and resources they need to navigate the ever-evolving digital terrain. By enhancing educators' digital competences and supporting their professional growth, the quality of education can be elevated, ensuring that students receive an education that prepares them for the challenges and opportunities of the digital age.

### Theoretical framework

Digital Teaching Competence (DTC) encompasses the knowledge, skills, and abilities required for effectively utilizing digital media. ^8^ It enables individuals to achieve various life goals through the use of technology. Consequently, the design, implementation, and execution of training initiatives for incorporating digital tools and pedagogical approaches in academic practice are considered essential professional requirements. ^9^

Furthermore, the integration of technology in education effectively depends on various factors related to the environment. ^10^ Therefore, it is crucial to acknowledge that DTC is a constantly evolving competence, parallel to the advancements in technology itself. ^11^ It is evident that DTC promotes learning when applied with interactive pedagogical tools, enhancing the efficiency of the theoretical model. ^12^

Within the framework of DTC, different dimensions are identified, categorized by models and standards established for its evaluation. The International Society for Technology in Education (ISTE) and the National Institute of Educational Technologies and Teacher Education (INTEF) propose five areas of digital competences for educators: information and information literacy, communication and collaboration, digital content creation, security, and problem-solving. ^13^^–^^15^ These competencies are further divided into three levels: Basic, Intermediate, and Advanced, which are classified as A1, A2, B1, B2, C1, and C2 based on the individual's level of proficiency.

It is worth noting that several studies conducted on DTC in the context of higher education reveal its transformative nature. The integration of digitalization in education presents two key tasks for professors: the development of their own digital skills and the cultivation of skills necessary for students to adapt to the digital world ^16^^,^^17^

Moreover, it is essential to recognize that digital competencies play a significant role in achieving the Sustainable Development Goals outlined in the United Nations' 2030 agenda. These competencies represent a set of skills that contribute to the pursuit of equality and social improvement.

Considering the aforementioned factors, it is important to highlight the assumed definitions of Digital Teaching Competence (DTC). It is described as the ability to develop operational capabilities with the support of various technological devices, enabling access to the internet for information retrieval. Consequently, the apprehension of DTC has emerged as a flexible and critical approach, adapting its use to the realities of the social environment, and evolving, expanding, and deepening its integration in all areas, including education. ^17^

Based on the literature reviewed, it is evident that the importance of DTC is recognized through the development of frameworks and self-evaluation instruments aimed at measuring the level of DTC appropriation by professors and educational managers, along with the integration and utilization of information and communication technology (ICT). ^18^^,^^19^ These frameworks and instruments align closely with educational policies and strategies proposed by Digitally Competent Educational Organizations, the European Framework for Digital Competence of Educators (DigCompEdu), the Mentoring Technology-Enhanced Pedagogy (MENTEP) project, and the United Nations Educational, Scientific and Cultural Organization (UNESCO). ^10^

On the other hand, UNESCO's Mentoring Technology-Enhanced Pedagogy Project (MENTEP) aims to incorporate the technical model of information and communication technologies (ICT) into the pedagogical environment to enhance teaching through technology, specifically through Massive Open Online Courses (MOOCs). This project also offers professors the opportunity to self-assess their digital competences and identify their areas of improvement through the TET-SAT standardized test ^20^

Additionally, the European Framework for Digital Competence of Educators (DigCompEdu) provides a reference for the development of digital competences at all educational levels. Based on a scientific foundation, DigCompEdu helps identify needs, deepen knowledge, expand skills, and promote professional development. It encompasses six competence areas: professional commitment, digital resources, teaching and learning, assessment and feedback, student empowerment, and development of students' digital competence. ^21^^,^^22^

Within the broad spectrum of instruments available to measure digital technological competencies (DTC), the selection of an appropriate tool is a critical process. This choice should not be based solely on criteria of robustness and reliability but must also consider its relevance to the specific research context. For this research, the DigCompEdu Check-in instrument was chosen. Originally designed by Ghomi and Redecker, this tool has undergone a rigorous translation and adaptation process led by Cabero-Almenara and Palacios-Rodríguez, thereby ensuring its applicability and relevance across various cultural and linguistic contexts.

What makes the DigCompEdu Check-in even more special, in addition to its meticulously standardized structure, is its open-access nature. This feature makes it a highly versatile tool that can be used in various studies and research contexts. It facilitates the assessment of a wide range of competencies and skills associated with the digital realm in education. Given its inherent robustness, reliability, and validity, it stands as a paramount choice in the research field, especially for studies centered on the deep understanding and analysis of educational digital competencies.

The decision to use this questionnaire is not based only on its outstanding intrinsic features but also on the significant recognition and trust it has accumulated within the scientific community. ^19^^,^^23^ Its open-access status not only underscores its adaptability but also highlights its contribution and value in the academic world, allowing for greater collaboration and consistency in research surrounding digital competencies.

In the current educational model, young people are immersed and actively engaged in the digital world. Therefore, ICTs serve as essential tools for their social and academic development. However, the use of technological devices, the internet, and social networks also exposes them to new challenges, dangers, threats, and electronic risks, such as harassment, identity theft, and scams.

Furthermore, the coronavirus disease 2019 (COVID-19) pandemic and the rapid transition to online learning worldwide have brought about significant transformations in teaching practices. ^24^ This situation raises questions about the preparedness of professionals for such changes and the digital capabilities of participants in the digitization process.

Given these considerations, it becomes necessary for professors to continuously update their knowledge regarding educational methods, pedagogical techniques, and technological advancements. Technology is recognized as one of the most influential tools across various domains, including social, educational, and professional spheres. Therefore, the teaching staff at the Technical University of Manabí (UTM) should actively engage in technological innovations, considering their direct and indirect involvement and commitment to society based on current educational realities.

It is noteworthy that while technologies continue to advance rapidly, professors often lag behind in keeping pace. This misalignment suggests that their actions may not always meet the demands of the globalized world. To address this, professors should actively seek ongoing training and development opportunities to cultivate broader capabilities. They should embrace new and motivating tools that facilitate different and innovative ways of learning for their students. ^25^ This involves employing teaching and learning methods and techniques that promote meaningful learning experiences.

Based on the aforementioned considerations, this study aims to achieve two objectives. Firstly, to analyze the self-perceived level of digital competence among professors at UTM in Ecuador. Secondly, to establish the relationship between age, sex, and academic profile variables and the digital competence of professors. The study adopts a quantitative approach, employing surveys to collect data based on sex, age, and level of academic training variables.

## Methods

To explore the proposed objectives, a descriptive correlational study was designed, supported by a quantitative approach and a non-experimental design structure. The methodological choice was neither random nor incidental but was deliberately aligned with the overall goals of the research. The focus was on delving into the essential core of the studied phenomenon and the intricate relationships between predetermined variables. ^26^ It is noteworthy that the strategy adopted is based on a strictly observational model, excluding any form of direct intervention or manipulation of the subject matter.

Within this framework, the following key elements are established:

• Critical phases in the research process were meticulously identified. These stages include the recruitment period for participants scheduled for the year 2022, specific moments for exposing the study subjects to the variables of interest, carefully structured follow-up phases, and predetermined timeframes for the empirical data collection.
• Proactive measures were taken to identify and mitigate potential sources of bias. For this, statistical methods such as random probability sampling and weighted tests were applied. These techniques were specifically adapted to the peculiarities and demands of the research with the aim of preserving objectivity and impartiality in the results.
• The selection of the number of participants was based on advanced statistical algorithms. These considered both the analytical power and the estimated effect size of the variables in question. This dual approach not only maximized the capability to detect significant interactions or impacts but also minimized the risks of erroneous inferences. In the event of missing data, consolidated statistical methodologies are applied. Initially, the nature and extent of the missing data were assessed. Subsequently, data imputation techniques were opted for, or alternatively, analyses based on complete records were carried out. This procedure ensured the integrity and reliability of the data and its subsequent interpretations.

### Ethical considerations

In accordance with the ethical imperatives governing academic research, a meticulous procedure for obtaining informed consent from participants was diligently executed prior to the administration of the research instrument. This is a critical facet in the realm of scientific inquiry, designed to ensure that participants are not only fully aware of the study's overarching aim and methodology but also of any prospective risks and benefits that may arise from their involvement.

To facilitate a comprehensive understanding of the study's parameters, informed consent documents were articulated in a lucid and accessible language, deliberately avoiding any complex technical terminology that could potentially obfuscate participants' comprehension of the study's scope and implications. This approach was adopted to reinforce the principle of voluntariness, emphasizing that participants were free to either abstain from or withdraw from the study at any point, without suffering any negative repercussions.

At the same time, strict protocols were established to safeguard the confidentiality and privacy of the data collected from the participants. Detailed explanations were provided regarding the mechanisms to protect the identity and personal data of the participants. Once any pending questions or concerns were addressed, participants were invited to officially register their consent. This was achieved through the signing of the informed consent document, before proceeding to administer the questionnaire through Google Forms.

The authorization for the execution of the current research was granted by the Institutional Ethics Committee, and the funding was facilitated by the Honorable University Council of the Technical University of Manabí. This support was institutionalized through the issuance of resolution RHCU.UTM-No.259-SO-10-2022, dated January 10, 2022, thus preceding the data collection phase.

### Participants and sampling

In the context of the current investigation, the designated study population consists of the full faculty body of the Technical University of Manabí, totaling 992 academic professionals (N=992). Through the deployment of a non-probabilistic, purposive, and voluntary sampling methodology, the research determined an estimated sample size of approximately 277 faculty members (n≈277). Unlike methods employing probabilistic sampling, this specific strategy did not aim to produce a representative subset of the population but focused on obtaining a more incidental selection of participants.

To facilitate the recruitment process, the researchers disseminated an electronic invitation to all faculty members at the Technical University of Manabí. This invitation included an embedded questionnaire tailored to the research objectives. Regarding the eligibility criteria, the researchers favored an expansive and inclusive approach, allowing participation from all faculty members, without imposing limitations based on departmental or disciplinary affiliations.

It is critical to acknowledge that due to the voluntary and non-probabilistic characteristics of the chosen sampling approach, the sample may not offer an accurate representation of the entire faculty demographic. However, the research team assessed this method as the most suitable, given the resource constraints and specialized objectives guiding the study.

In an effort to obtain a comprehensive range of perspectives relating to digital competencies, the study included faculty members from various academic departments and diverse professional backgrounds. This consideration of multidimensional diversity gains particular relevance in the context of the research objectives, which aim to explore the dimensions of digital competencies within the unique academic environment of the Technical University of Manabí.

To conclude, it is essential to underscore that electronic mail served as the primary channel for the distribution of research questionnaires. This approach not only streamlined the data collection process but also provided an effective mechanism for preserving participant anonymity and convenience. These attributes conformed to the ethical and logistical requisites integral to the research design.

### Data collection instrument

In the current research, the DigCompEdu Check-in instrument was used for data collection. ^41^ This tool was initially developed by Ghomi and Redecker in 2019 and disseminated by the Joint Research Center. To meticulously adapt it to the Spanish context, Cabero-Almenara and Palacios-Rodríguez ^23^ stepped in, ensuring its availability in open access and under a Creative Commons Recognition License for its use in various research endeavors. The instrument is structured as a survey and encompasses 22 items, which are categorized into six competency domains: Professional Commitment, Digital Resources, Digital Pedagogy, Assessment and Feedback, Empowering Students, and Facilitating Students' Digital Competence. A five-interval Likert scale was utilized to evaluate the responses, offering five answer options for each item. Additionally, demographic variables such as gender and age were collected.

The coding of the items was accomplished using an alphanumeric combination of two or three letters. In the case of competence domains denoted by two letters, the first letter signifies the particular domain. For example, the letter “C” is associated with the “Professional Commitment” domain, while the subsequent letters, ranging from ‘A’ to ‘D,’ identify specific items within that domain. This coding pattern is replicated in other domains, using, for instance, the letter “R” for the “Digital Resources” domain and “P” for “Digital Pedagogy.”

As for the administration method, the survey was distributed via Google Forms. This digital tool not only facilitated efficiency in data distribution and collection but also ensured the anonymity of the participants. In this way, ethical guidelines were met, and logistical considerations were addressed, thus ensuring the appropriate implementation of the research design.

### Data analysis

Following the data collection via surveys, an inferential analysis was conducted with the aim of elucidating the self-perception that educators have regarding their digital competencies. To eliminate ambiguities in the categorization of these competencies, a secondary alphabetical character was assigned to each group when two groups shared the same initial letter. Thus, “Evaluation and Feedback” was coded as “EfV,” while “Empowerment” was labeled as “EP.”

During the variable construction phase, a composite index (CALIF) was calculated for each participant. This was achieved by summing the corresponding values of the responses for the items linked to each competency domain. The composite index ranged from 0 to 88 points. Once these scores were obtained, the individuals' competency levels were categorized according to the grading scheme outlined in Table 1.

**Table 1.** Classification and scoring system of the “DigCompEdu Check-In” competence level.

Level of competence	Score (out of 88 points)
Novice (A1)	<20
Explorer (A2)	20-33
Integrator (B1)	34-49
Expert (B2)	50-65
Leader (C1)	66-80
Pioneer (C2)	>80

Source ^23^.

The processing and analysis of the data were carried out using the SPSS-21 software, a tool that ensures solidity and accuracy in the achieved results. Despite the advantages offered by this licensed software, it is prudent to consider open-source alternatives, such as JASP, which is not only free but also features a graphical interface for statistical analysis.

For each area of competence, a variable was generated with the sum of the scores of the items that constituted the area of competence, and together with each numerical variable, a categorical variable was conceived as suggested by, ^23^ as specified below:

COMMITMENT. Based on the responses of the competence areas “Professional Commitment”, it took values from 0 to 16 points, its categorical variable was named COMP_C.

DIGPEDAGOGY. Based on the responses of the competence areas “Digital pedagogy”, which took values from 0 to 16 points, its categorical variable was named PEDAGO_C.

DIGRESOURCES. Based on the responses of the competence area “Digital Resources”, which took values from 0 to 12 points, its categorical variable was named RECDIGC.

EMPOWER. Based on the responses of the competence area “Empowering Students”, which took values from 0 to 12 points, its categorical variable was labeled EMPODERAC.

EVALUATESANDPRO. Based on the responses of the competence area “Evaluation and Provides feedback”, which took values from 0 to 12 points, its categorical variable was labelled EVALUAYRC.

FACILITATESCO. Based on the competence area, “Facilitating Students' Digital Competence”, which took values from 0 to 20 points, its categorical variable was named FACILITACOC.

The validation of the instrument was carried out through the reliability test through Cronbach's Alpha, where it could be determined in a general and internal way, applying for this purpose the SPSS-21 statistical software, as shown in Table 2. Likewise, 22 items were used giving a value of 0.949, indicating a high level of reliability. Table 3 includes the result of the behavior of each item to the reliability, which showed the importance of all the items within the instrument.

**Table 2.** Reliability statistics.

Cronbach's Alpha	N of elements
0.949	22

**Table 3.** Cronbach's reliability test results.

Area	Competence	Scale mean if the item has been deleted	Scale variance if the item has been deleted	Correlation total corrected items	Cronbach's alpha if the item has been deleted
Professional Engagement	Organizational Communication	52.5343	129.018	0.500	0.949
	Professional Collaboration	52.8448	128.885	0.611	0.948
	Reflective Practice	52.5812	127.708	0.627	0.947
	Digital Training	51.9458	124.283	0.654	0.947
Digital Resources	Selection	52.5668	128.609	0.596	0.948
	Creation and Modification	52.5307	129.388	0.623	0.948
	Administration, Sharing, and Protection	52.3827	126.686	0.607	0.948
Digital Pedagogy	Teaching	52.4585	125.698	0.694	0.947
	Guidance	52.1913	127.510	0.703	0.946
	Collaborative Learning	52.2888	128.699	0.640	0.947
	Self-directed Learning	52.2166	129.272	0.669	0.947
Evaluation and Feedback	Evaluation Strategies	52.2635	128.166	0.702	0.947
	Analysis of Evidence and Tests	52.3863	126.252	0.716	0.946
	Feedback and Planning	52.6643	127.840	0.723	0.946
Empowering Students	Accessibility and Inclusion	52.1047	128.029	0.642	0.947
	Differentiation and Personalization	52.4693	126.235	0.677	0.947
	Active Student Participation	52.3899	125.492	0.751	0.946
Facilitating Students' Digital Competence	Information and Media Literacy	52.5235	127.316	0.689	0.947
	Digital Communication and Collaboration	52.4513	127.401	0.700	0.947
	Creation of Digital Content	52.2491	127.115	0.718	0.946
	Responsible Use and Well-being	52.4801	126.555	0.735	0.946
	Digital Problem Solving	52.4296	127.688	0.653	0.947

## Results

This section presents the findings of this study. ^40^ First, a reliability test was conducted using Cronbach's alpha coefficient to assess the internal consistency of the survey items (see Table 3). Cronbach's alpha is a widely used measure to evaluate the reliability of a scale or questionnaire. It indicates the extent to which the items in the instrument measure the same underlying construct.

Table 3 displays the results of the reliability test. The coefficient value obtained provides an indication of the overall internal consistency of the instrument. A higher value suggests a greater degree of reliability, indicating that the items in the instrument measure the construct consistently. Conversely, a lower value may indicate inconsistency in the measurements.

The reliability test was conducted using statistical software, such as SPSS-21, which allows for the calculation of Cronbach's alpha coefficient. The test involved analyzing the responses to the 22 items included in the instrument. Reliability statistics provide valuable information about the robustness and stability of the instrument used in the study. By evaluating the internal consistency of the items, researchers can ensure that the instrument is reliable and yields consistent results.

Table 3 delineates the outcomes of Cronbach’s alpha reliability assessment for a range of competencies, segmented under various domains pertinent to professional endeavors. These domains encompass Professional Engagement, Digital Resources, Digital Pedagogy, Evaluation and Feedback, Empowering Students, and Facilitating Students' Digital Competence.

Upon meticulous examination, it becomes evident that all competencies under investigation exhibit remarkable reliability. This assertion is substantiated by Cronbach’s alpha values, which span between 0.946 and 0.949. Such values, particularly when surpassing the 0.7 threshold, are conventionally deemed to be emblematic of substantial internal consistency.

Transitioning our focus to the scale mean and variance in the event of item deletion offers a profound understanding of the potential fluctuations in these metrics should a specific competency be excluded. On average, the scale mean gravitates around 52.5 for the various competencies, insinuating a harmonious scale. In parallel, the scale variances, which oscillate primarily between the mid-120s and 130, display marginal variations among the competencies, reinforcing the notion that there are no pronounced disparities among them.

Moreover, an analysis of the correlations unveils a discernible linear association between individual competencies and the corrected total score. This score represents the cumulative value of all items, excluding the item currently under consideration. Notably, the correlation coefficients span from 0.500, evident in the Organizational Communication within the Professional Engagement domain, to 0.751 observed in Active Student Participation under Empowering Students. These consistently positive correlations imply that an uptick in scores for individual competencies is frequently associated with a surge in the overall score.

Lastly, by evaluating the column that depicts Cronbach's alpha in scenarios where an item has been excised, one gleans pivotal insights regarding the influence of individual competencies on the overarching reliability of the measurement tool. An attenuated value in this column, in contrast with the intrinsic Cronbach's alpha, intimates a potential decrement in reliability upon the removal of the said competency. Nonetheless, the relatively minute distinctions underscore that any singular competency's omission would unlikely induce a substantial shift in the tool's internal consistency.

Subsequently, a descriptive analysis was conducted to examine the participants' responses, considering the five intervals established by the Likert scale ( Table 4). Descriptive analysis involves summarizing and interpreting the collected data to provide a comprehensive understanding of the participants' evaluations.

**Table 4.** Results of the descriptive analysis on the areas of digital competence of professors (n=277).

	Categories					
Competence	Novice	Explorer	Integrator	Expert	Leader	Pioneer
PROFESSIONAL COMMITMENT	3.25	16.97	36.46	38.99	3.61	0.72
RECOGNIZES EVALUATES AND EMPOWERS	2.53	13.00	39.35	35.38	9.03	0.72
DIGITAL PEDAGOGY	0.36	9.39	40.07	39.71	7.94	2.53
EVALUATES AND PROVIDES FEEDBACK	1.44	9.03	46.21	32.13	7.58	3.61
EMPOWERS STUDENTS	2.53	8.30	32.85	42.60	9.03	4.69
FACILITATES COMPETENCES	2.53	5.05	48.74	36.82	3.61	3.25

Source: Data provided by the respondents, ^40^ processed with SPSS-21 statistical software.

The results presented in Table 4 highlight the distribution of participants across different competence levels in each area. In the Professional Commitment competence, the highest levels are observed among experts and integrators, accounting for 38.99% and 36.46% respectively. On the other hand, the pioneer level has the lowest representation with only 0.72%, followed by the explorer, leader, and novice levels, which account for 16.97%, 3.61%, and 3.25% respectively.

Turning to the Recognizes, Evaluates and Empowers competency, Table 4 reveals that the integrator category holds the highest percentage with 39.35%, followed by the expert category with 35.38%. The explorer category represents 13.00%, while the novice, leader, and pioneer categories account for 2.53%, 9.03%, and 0.72% respectively.

In the Digital Pedagogy competence, the integrator category stands out with the highest representation at 40.07%, closely followed by the expert category at 39.71%. The explorer category accounts for 9.39%, while the leader, pioneer, and novice categories have percentages of 7.94%, 2.53%, and 0.36% respectively.

In the Evaluates and Provides Feedback competence, 46.21% of the surveyed professors position themselves in the integrator category, while 32.13% identify themselves as experts. Additionally, 9.03% consider themselves explorers, 7.58% as leaders, 3.68% as pioneers, and 1.44% as novices.

Regarding the Empowers Students competence, the results indicate that 42.60% of the respondents have an expert level, while 32.85% are classified as integrators. The leader, explorer, pioneer, and novice categories represent 9.03%, 8.30%, 4.69%, and 2.53% respectively.

Examining the Facilitates Competences competence, the majority of professors, 48.74%, are categorized as integrators, followed by experts at 36.82%. The explorer, leader, pioneer, and novice categories account for 5.05%, 3.61%, 3.25%, and 2.53% respectively.

These results provide insights into the distribution of participants across competence levels, highlighting the variations in self-perceived digital competences among the surveyed professors. Understanding these distributions is crucial for designing targeted interventions and support strategies to enhance digital competences and promote professional development in the educational context.

Table 5 presents the analysis of variance, which examines the sources of variation, the degrees of freedom, and the sum of squares for each numerical variable investigated. Analysis of variance (ANOVA) is a statistical technique used to determine the significance of differences between groups or categories.

**Table 5.** Summary of variance analyses.

Source of variation	gl	Commitment	DigResources	Digital pedagogy	Empowers	Evaluatesandpro	Facilitatesco
Sex	1	0.194	11.978	3.956	0.561	3.168	14.771
Academic profile	3	13.812	6.320	4.432	5.814	0.963	10.764
Age	3	5.725	7.215	30.762	20.350	16.314	59.216
Sex × academic profile	3	42.867	23.227	**46.227**	17.368	11.432	43.846
Sex × Age	3	31.892	9.857	15.598	6.605	6.975	27.227
Academic profile × Age	5	11.394	6.288	13.966	17.036	8.279	32.639
Sex × academic profile × Age	2	8.429	11.755	8.799	3.864	2.409	2.676
Error	257	1635.739	857.105	1386.356	1039.333	880.300	2313.342
**Total corrected**	277	1805.726	954.671	1500.686	1104.801	932.108	2533.199

Source: Data provided by respondents, ^40^ processed with SPSS-21 statistical software.

When examining the sources of variation such as sex, academic profile, and age, as well as their interactions, significant differences are observed in the “pedagogy variable” due to the interaction effect between sex and academic profile. This suggests that the relationship between sex and academic profile influences the variability in pedagogical competence.

Furthermore, the relationship between academic profile and age accounts for approximately 31.892% of the variability in professional commitment. This indicates that the combination of academic profile and age significantly contributes to variations in the level of professional commitment.

Similarly, the confluence of sex, academic profile, and age has a notable impact, accounting for 11.755% of the variability in digital resources. This suggests that the interaction among these factors plays a significant role in determining the level of digital resource competency.

Table 6 provides a summary of the Chi-Square test results, which examines the independence of each factor against all categorical variables in each competence area. The “Pvalue” column indicates the significance of each test, with a significance criterion of α=0.05. If the test is not significant, it suggests that the variables are independent. However, for example, in the case of the “Evaluates and provides feedback” variable, a significant relationship with sex is found.

**Table 6.** Chi-square test for the factors of sex, age and academic education level.

Factor	Levels	Chi squared	Gl	Pvalue
**Sex**	Professional commitment	3.767 ^a^	5	0.583
	Digital pedagogy	4.983 ^a^	5	0.418
	Recognizes, evaluates	6.142 ^a^	5	0.293
	Evaluates and provides feedback	15.543 ^a^	5	0.008
	Empowers students	7.204 ^a^	5	0.206
	Facilitates competences	1.903 ^a^	5	0.862
**Age**	Professional commitment	29.113 ^a^	15	0.016
	Digital pedagogy	32.688 ^a^	15	0.005
	Recognizes, evaluates	36.243 ^a^	15	0.002
	Evaluates and provides feedback	24.878 ^a^	15	0.052
	Empowers students	15.910 ^a^	15	0.388
	Facilitates competences	42.415 ^a^	15	0.0001
**Academic profile**	Professional commitment	15.540 ^a^	15	0.413
	Digital pedagogy	24.410 ^a^	15	0.058
	Recognizes, evaluates	9.339 ^a^	15	0.859
	Evaluates and provides feedback	20.957 ^a^	15	0.138
	Empowers students	28.461 ^a^	15	0.019
	Facilitates competences	16.066 ^a^	15	0.378

Source: Data provided by respondents, processed with SPSS-21 statistical software.

Regarding age, independence is observed in the “Evaluates and provides feedback” and “Empowers students” variables, suggesting that age does not significantly affect these competences. However, in the other variables, a significant relationship is found between age and the rest of the competences, indicating a non-independent effect of age on those variables.

For the academic profile, independence is found in relation to age, except in the “Empowers students” variable. This implies that the academic profile has a significant relationship with age in most competences, except in the case of empowering students.

These findings highlight the complex interplay of factors and their interactions in influencing the variations observed in different competence areas. Understanding these relationships is essential for tailoring educational interventions and designing targeted strategies to enhance specific competences based on sex, academic profile, age, and their combinations.

**Figure 1.**
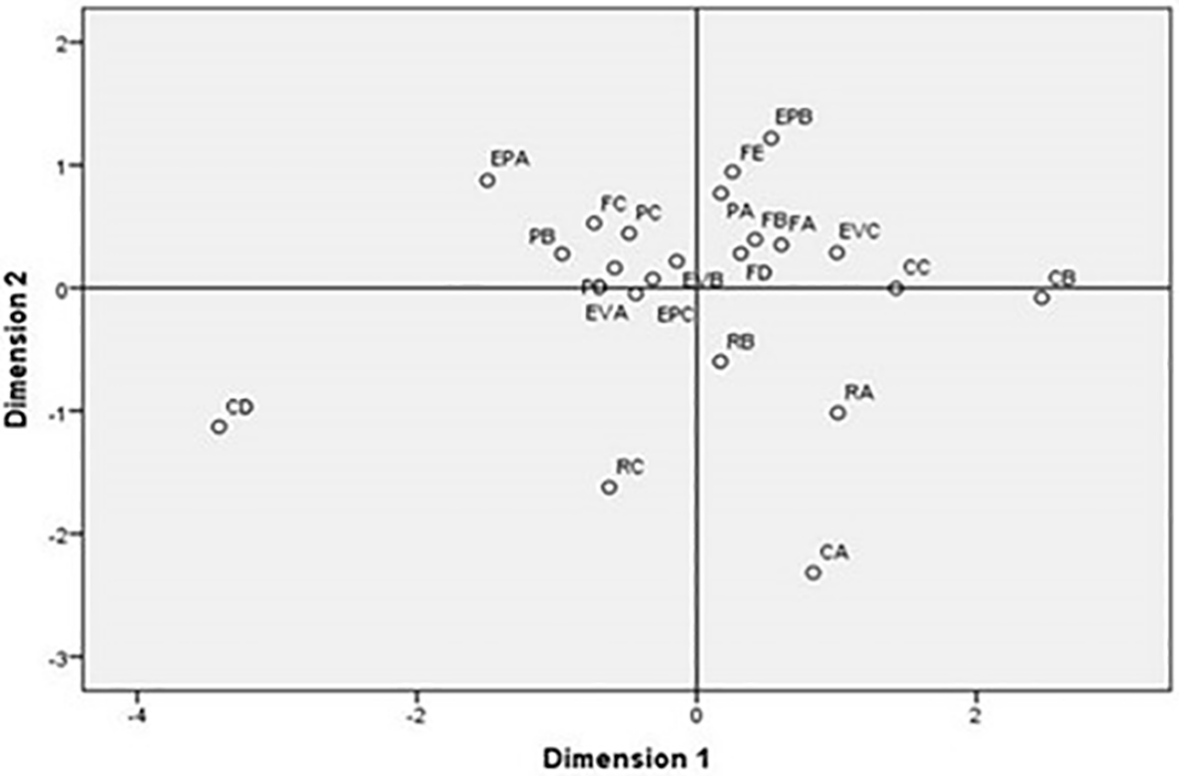
Scaling of competences. Source: Data provided by respondents, processed with SPSS-21 statistical software.

Within the possibilities of multidimensional scaling with the application of the multivariate technique, stress is presented as a measure of goodness of fit, whose indicator was ‘weak to poor’ with a value of 0.1828, which is reflected in the graph represented by a regression in Figure 1, where the degree of coupling of the data can be observed. However, in the Stress value, it was visualized that there was closeness between the items of the ‘Empowers’, ‘Evaluates’, ‘Facilitates Digital Competence’ competence areas. Therefore, the items of these areas of competence formed two compact groups of items, sharing items between one group and the other, but indicating that in general these items formed a construct.

As it can be observed **,** it was verified that Professional Commitment and Digital Resources were left out of this group. In this way, it was observed that the most dispersed items were those of Professional Commitment. On the other hand, only “Reflective Practice” and “Professional Collaboration” were relatively close to each other, but very far from “Digital Training” and “Organizational Communication”. Likewise, the “Digital Resources” items were found to be close to each other. Consequently, it could be considered that the items of these two areas formed another construct.

Next, a two-stage factor analysis was performed.

The results of the factor analysis with the maximum likelihood method and the “Oblimin” oblique rotation method option showed a KMO value of 0.961 and Bartlett's test of sphericity was significant, indicating that the application of the factor analysis was adequate.

The lower part of Table 7 shows the characteristic values of the first two factors, and it was observed that with the first two factors a cumulative explained variance of 56.58% was obtained which was considered acceptable, suggesting a two-factor model. The first factor was made up of the competences that were mainly aimed at the achievement of empowerment by students of digital tools to apply them to the teaching-learning process.

**Table 7.** Factor analysis of items.

Area/Competence	Factor	
	1	2
Facilitating Students' Digital Competence/Digital Communication and Collaboration	0.859	
Facilitating Students' Digital Competence/Responsible Use and Well-being	0.809	
Empowering Students/Differentiation and Personalization	0.800	
Facilitating Students' Digital Competence/Digital Problem Solving	0.788	
Facilitating Students' Digital Competence/Creation of Digital Content	0.787	
Facilitating Students' Digital Competence/Information and Media Literacy	0.702	
Evaluation and Feedback/Feedback and Planning	0.659	
Evaluation and Feedback/Analysis of Evidence and Tests	0.631	
Digital Pedagogy/Collaborative Learning	0.597	
Empowering Students/Accessibility and Inclusion	0.595	
Empowering Students/Active Student Participation	0.590	
Digital Pedagogy/Self-directed Learning	0.472	
Digital Pedagogy/Guidance	0.461	
Digital Pedagogy/Teaching	0.393	0.373
Professional Engagement/Digital Training		0.783
Digital Resources/Selection		0.678
Digital Resources/Creation and Modification		0.640
Professional Engagement/Organizational Communication		0.633
Professional Engagement/Reflective Practice		0.442
Evaluation and Feedback/Evaluation Strategies	0.368	0.409
Digital Resources/Administration, Sharing, and Protection		0.373
Auto value	10.862	1.285
% Variance explained	49.374	5.840
% of cumulative variance	49.374	55.214

Source: Data provided by respondents, processed with SPSS-21 statistical software.

The second factor generated a construct that described the professors’ personal attitudes toward their training and use of digital tools. That is, construct one referred to the entire pedagogical structure, and construct two to the professors’ personal training and use of it.

Similarly, Table 7 presents the factors and the loadings of each of the items within the factors. Thus, the rotation analysis made it possible to achieve what the literature suggests as a good result in factor analysis, in other words, that each factor is made up of variables with high values and some variables with values close to zero, and that each variable belongs to only one of the factors.

Therefore, variables with small values in the factors were omitted to avoid duplicities, taking as a criterion for the elimination of values lower than 0.35. For this reason, only two items could not be fully discriminated against, which were Professional Collaboration and Evaluation Strategies. However, Professional Collaboration was more important in factor 1, while Evaluation Strategies was more important in factor 2. Finally, it was observed that the item Digital Training was not part of any of the factors to obtain the scores.

**Figure 2.**
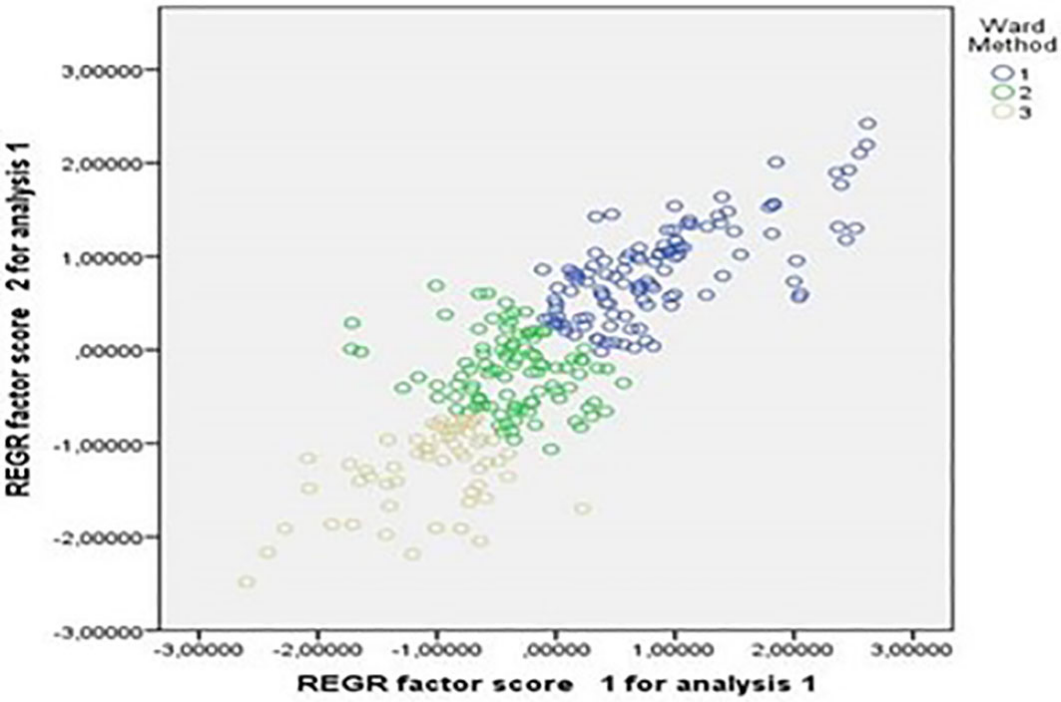
Assisted clustering analysis of the constructs. Source: Data provided by respondents, processed with SPSS-21 statistical software.

It is clear that through the assisted clustering analysis, three groups were identified. These are shown in Figure 2.

In this analysis, it was found that the grouping of participants was strongly influenced by factor 1, which pertained to the pedagogical aspect. Group 1 comprised professors who exhibited higher ratings on construct 1, indicating a strong emphasis on pedagogy. Group 2 represented an intermediate category, characterized by a balance between the two constructs. On the other hand, Group 3 consisted of professors whose ratings on construct 2 prevailed.

These groupings suggest that participants varied in their emphasis on different aspects of teaching and learning. Group 1 highlighted a strong focus on pedagogical approaches, while Group 3 demonstrated a greater emphasis on the second construct, which may be related to other dimensions or competences.

Understanding these groupings provides insights into the diverse perspectives and preferences of educators in relation to teaching practices and competences. It enables researchers and educators to identify specific areas where professional development and support can be targeted to enhance teaching effectiveness and promote a well-rounded approach to education.

To describe more precisely the constitution of the groups, the means corresponding to each of the variables that calculated the score in each area of competence were generated, the results of which are shown in Table 8.

**Table 8.** Means and standard deviation of the groups formed.

Variable	G1 (n=117)		G2 (n=101)		G3 (n=59)	
	Mean	Standard deviation	Mean	Standard deviation	Mean	Standard deviation
COMMITMENT	11.85	1.428	9.0792	1.56002	6.509	1.569
DIGRESOURCES	8.69	1.296	6.8119	1.14641	5.034	1.144
DIGPEDAGOGY	12.35	1.698	9.8119	1.42627	7.797	1.200
EMPOWER ESTUD	9.29	1.327	7.1683	1.49713	5.661	1.360
FACILITATESCO	14.86	2.297	11.1782	1.83519	9.509	1.995
EVALUATES AND PROVIDES FEEDBACK	8.85	1.538	6.7426	1.00651	5.610	1.145

Source: Data provided by respondents, processed with SPSS-21 statistical software.

To provide a more detailed description of the group composition, the means for each variable that contributed to the calculation of the score in each competence area were computed. The results of this analysis are presented in Table 8.

Table 8 displays the mean values for each variable within each competence area. These means offer a comprehensive understanding of the specific aspects or dimensions that contribute to the overall score in each competence area. By examining the means, researchers can identify the relative strengths and weaknesses of the participants in different areas of competence.

Analyzing the means enables a more nuanced interpretation of the group composition and allows for a deeper exploration of the participants' competences. It provides valuable insights into the specific areas where educators excel or require further development.

By considering these mean values, researchers and educators can design targeted interventions and strategies to enhance specific competences and address any areas of improvement identified. This information contributes to the overall goal of fostering professional growth and enhancing the quality of teaching and learning experiences.

## Discussion

Upon examining the results, it is evident that the Integrator and Expert categories consistently present higher percentages in various competencies, such as PROFESSIONAL COMMITMENT, RECOGNIZE, EVALUATE AND EMPOWER, DIGITAL PEDAGOGY, EVALUATE AND PROVIDE FEEDBACK, EMPOWER STUDENTS, and FACILITATE COMPETENCIES. This indicates that teachers who perceive themselves as integrators and experts demonstrate a higher level of Digital Teaching Competence compared to explorers and novices. Additionally, leaders and pioneers, albeit fewer in number, show greater knowledge and abilities in these competencies.

These findings align with studies conducted by Espino-Díaz *et al.,* ^27^ and Hämäläinen *et al*. ^28^ which similarly highlight a pattern of behavior in competencies leaning towards a low to medium competence level. The study by Peled ^29^ also supports this idea, suggesting that there is a positive correlation between teachers' self-perception as experts or integrators and their level of Digital Teaching Competence.

In terms of demographic factors, Table 5 shows that sex and academic profile exhibit the highest values on the digital pedagogy variable. This suggests that, regardless of sex, educational teaching techniques are applied without gender bias, contributing to the development of Digital Teaching Competence. Furthermore, the lower values imply that sex is not a significant factor in acquiring commitments or fulfilling professional responsibilities in teaching.

These findings align with the discoveries of Guillén-Gámez *et al.,* ^30^ who emphasize the limited differences between sex and age in terms of self-perception of digital competencies. This corroborates that sex does not have a significant impact on learning and teaching abilities related to the use and management of digital tools. It also suggests the need to plan and implement strategies for integrating digital competencies into teacher training programs.

Regarding age, the percentages indicate that it is not perceived as a limitation for acquiring or fulfilling commitments related to the appropriation of digital resources. This finding aligns with Suárez and Colmenero, ^31^ where no statistically significant association was found between age and competence level, indicating that there are no significant differences among different age groups. This result is also backed by the study of Gudmundsdottir and Hatlevik, ^32^ suggesting that digital competence is not determined by age, but rather by attitude and training.

Table 6 examines the relationship between factors, including sex, age, and academic profile. It indicates that sex is independent of the variables, except for “Evaluate and provide feedback,” suggesting that sex influences the search for possibilities to identify and verify knowledge. Also, the “Evaluate and provide feedback” and “Empower students” variables show the lowest values, indicating that age does not determine processes involving communication with students.

Regarding the academic profile, the “Empower students” variable obtains the highest score, indicating that teachers strive to provide students with the necessary support and information to guide their development. Although some teachers possess basic or intermediate digital skills, it is essential that they empower their students through adequate training. This aligns with the study conducted by Colás-Bravo *et al*., ^33^ emphasizing the need for teachers to contribute to developing critical, reflective, creative, and innovative thinking in students through proper training. Aidoo *et al.* ^34^ study supports this idea, suggesting that teachers need to have a deeper understanding of how to use digital tools to empower students and foster their autonomous learning.

The correlation analysis presented in Figure 1 highlights the interrelationship between items related to competencies such as “Empower”, “Evaluate”, and “Facilitate Digital Competence.” This suggests that as teachers set out to empower students, they contribute their own competencies to improve students' skills, thus demonstrating their commitment to professional performance and teaching praxis.

However, it is important to note that Professional Commitment and Digital Resources were not included in this construct, indicating that not all teachers share the same attitude towards student training, possibly due to resource limitations. Another construct formed by “Digital Training” and “Organizational Communication” suggests the existence of divided positions regarding empowerment practices.

Findings from List ^35^ support this idea, as they indicate that the management of digital identities in the educational context, specifically in the dimensions of communication and collaboration, is limited among teachers. This suggests that there is a need for further development of digital competencies among educators. Similarly, the study of Benitt *et al.* ^36^ concludes that teachers need more comprehensive training in digital competencies to effectively utilize technologies in teaching.

Table 7 provides an analysis of the factors, indicating that Professional Collaboration competencies, which contribute to the application of strategies in adapting virtual environments, have greater relevance in educational processes. Also, Evaluation Strategies is identified as a significant factor in determining the extent of learning and organizing improvements. This aligns with the study conducted by López-Belmonte *et al.,* ^37^ emphasizing the importance of promoting teachers' participation in training programs focused on developing digital competence and using emerging methodologies. These programs aim to adapt teaching practices to the new techno-pedagogical paradigms of a digitized society.

Figure 2 identifies three distinct groups of teachers. The first group (G1) emphasizes the importance of grades in pedagogical processes, while the second group (G2) lacks a clear definition. The third group (G3) expresses limited contributions to the topic. These findings are consistent with the conclusions drawn by Gil-Jaurena and Domínguez, ^22^ who recommend incorporating more profound educational technology content into training programs to improve educational quality and enable appropriate teaching praxis for the digital age. Moreover, the findings of the study by Rapanta *et al* ^38^ support this recommendation, suggesting that a better understanding of educational technology can help teachers adapt their practices to students' needs and expectations in the digital environment.

Finally, Table 8 presents the mean values and standard deviations of the formed groups. It reveals that Group 1 (G1) shows the lowest proportional effect on the “DIG RESOURCES” variable, indicating that without adequate digital resources, teachers face limitations in providing quality education. On the other hand, Group 1 (G1) shows the highest weighted average on the “FACILITATESCO” variable, suggesting that the ability to facilitate required competencies largely depends on the interaction between teachers and their students. In Group 3 (G3), the “DIGRESOURCES” variable receives the lowest scale, underlining the importance of having digital resources to promote skills for effective use of digital environments. These findings align with the study conducted by Pozo-Sánchez *et al*., ^39^ which associates higher digital competence scores with dimensions related to information and communication literacy and collaboration.

The study provides a comprehensive view of how teachers perceive themselves in terms of digital competence and how this perception relates to demographic variables such as gender, age, and academic profile. However, certain limitations and ambiguous areas suggest the need for further and more detailed research in the future.

## Conclusions

The findings of the descriptive analysis shed light on the self-perception of digital teaching competence among UTM professors, revealing an overall trend towards the integrator and expert categories. Notably, competences such as “Facilitates Competences,” “Evaluates and Provides Feedback,” and “Digital Pedagogy” were frequently highlighted.

Furthermore, the study identified significant differences in the relationship between age, sex, and academic profile and professors' digital competence, particularly in the “Digital Pedagogy” domain. This indicates an inconsistent relationship between sex and academic profile, with most variables exhibiting independence, except for the “Evaluates and Provides Feedback” variable, which showed significance.

Regarding age, a relationship was found in the “Evaluates and Provides Feedback” and “Empower Students” variables, while independence was observed for the remaining variables. The study acknowledges certain limitations, primarily the lack of random sampling, which hindered the creation of a more balanced and homogeneous sample. Consequently, the generalizability of the findings may be limited.

Future research directions should focus on designing training programs tailored to address the specific digital teaching competence needs of professors in order to effectively tackle the challenges posed by the digital era. These programs should incorporate formative and procedural aspects that allow participants to actively produce and implement their learning, emphasizing practical application to meet real needs.

## Data Availability

Figshare: Data- Digital teaching competence of higher education professors.xlsx. https://doi.org/10.6084/m9.figshare.24084393. ^40^ The project contains the following underlying data:
- Data- Digital teaching competence of higher education professors.xlsx Data are available under the terms of the Creative Commons Zero “No rights reserved” data waiver (CC0 1.0 Public domain dedication). Figshare: European Framework for Digital Competence of Teachers (DigComEdu) questionnaire. https://doi.org/10.6084/m9.figshare.24224065. ^41^ The project contains the following extended data:
- QUESTIONNAIRE (DigComEdu).xlsx Data are available under the terms of the Creative Commons Attribution 4.0 International license (CC-BY 4.0).
