# Digital teaching competence of higher education professors: self-perception study in an Ecuadorian university

**DOI:** 10.12688/f1000research.139064.2

**Published:** 2024-10-29

**Authors:** Jenniffer Sobeida Moreira-Choez, Jimmy Manuel Zambrano-Acosta, Alexander López-Padrón

**Affiliations:** 1 Postgraduate Institute, Technical University of Manabí, Portoviejo, Manabí, 130101, Ecuador

**Keywords:** Digital competences, university professors, self perception, educational modality, information and Communication Technologies

## Abstract

**Background:**

Teaching professionalization aimed at the digital transformation of educational scenarios and training processes for students in contemporary higher education requires the mastery of digital competence by the teaching staff. The objectives of the study were to analyze the self-perceived level of digital teaching competence (DTC) of the faculty of the Technical University of Manabí (UTM), Ecuador, and to establish the relationship between age, sex, and academic profile variables with digital teaching competence.

**Methods:**

A quantitative methodological approach was adopted to develop a descriptive-correlational field study with a non-experimental design. The participants were 277 professors, selected through non-probabilistic and voluntary sampling, who completed the DigCompEdu Check-In questionnaire sent by e-mail.

**Results:**

The data revealed that the “integrator” and “expert” categories achieved high levels in all competencies. In particular, 48.74% of the participants were placed in the integrator category in the competence of facilitating skills, while 46.21% positioned themselves as integrators in the competence of evaluation and feedback. Additionally, a significant difference was found in the pedagogy variable in the interaction.

**Conclusions:**

It is concluded that the competences self-perceived by the professors are within the intermediate categories such as integrator and expert. Likewise, the age, sex, and academic profile variables differ in the digital pedagogy level, which produces an inconsistent relationship, with the exception of the variable evaluates and provides feedback, where it was significant.

## Introduction

Technology plays a crucial role in contemporary and future society, with significant implications for the educational field. ^1^ The rapid pace of technological change has introduced profound challenges in teaching and learning. ^2^ As a result, educators must recognize the necessity of self-directed professional development to enhance their expertise, including achieving an adequate level of digital literacy. This proficiency is essential for success across various dimensions of education and training. ^3^

As key institutions responsible for fostering the development of professional competence, universities must possess qualified resources, both in terms of materials and human capital, to prepare well-rounded professionals. ^4^^,^^5^ These professionals should be equipped with problem-solving skills, critical thinking abilities, and the right attitudinal disposition necessary to navigate the complexities of the digital age. ^6^^,^^7^ However, despite the acknowledged importance of technology in education, there are significant gaps in research regarding how educators can effectively select and utilize technological tools within their teaching practices.

Therefore, it becomes crucial for educators to develop proficiency in selecting appropriate technological tools and seamlessly integrating them into their instructional approaches. ^8^^,^^9^ By doing so, they can plan and implement activities that foster learning environments conducive to student success. ^10^ Unfortunately, there is a dearth of comprehensive research that explores how educators can fully leverage the potential of technology to enhance teaching and learning in the classroom.

Consequently, it is essential for educators to develop competencies in the proper selection of technological tools and their effective integration into pedagogical approaches. ^11^^,^^12^ This skill enables the planning and implementation of activities that promote learning environments conducive to student success. However, there is a noticeable lack of comprehensive research exploring how educators can fully harness the potential of technology to enhance teaching and learning processes in the classroom. ^13^^–^^15^

The findings of this research will have significant implications for educational institutions, policymakers, and educators themselves. ^16^ Understanding educators’ self-assessment of their digital competencies will provide clear insight into their level of confidence and awareness regarding the use of technology in education. ^17^ Additionally, identifying the challenges they face in selecting and utilizing technological tools will offer valuable insights into the barriers that hinder the effective integration of technology into their pedagogical practices.

In light of this situation, this study aims to address these research gaps by examining educators’ self-assessment of their digital competencies and their ability to effectively select and utilize technological tools in their teaching practice. Understanding the challenges and needs faced by educators in this context will enable the development of more effective training and support strategies, ^18^ contributing to their professional development and enhancing the quality of education in the digital age. ^19^

In this context, the following question arises: What level of preparedness do the faculty members at the Technical University of Manabi (UTM) have in managing digital tools? To answer this question, two essential objectives are set. The first is to analyze the self-perceived level of digital competence (DC) among the faculty at UTM. The second is to establish the relationship between the variables of age, gender, and academic profile with this digital competence. The study was conducted using a quantitative approach, applying surveys to the faculty, considering gender, age, and academic background as variables. These factors were analyzed to identify potential correlations and establish patterns that contribute to a better understanding of the digital preparedness of faculty members in the current educational context.

### Theoretical framework

As education increasingly moves towards digitalization, teachers’ digital competences have become crucial for ensuring teaching quality and effectiveness. The integration of technology into pedagogical processes demands continuous training and adaptation from educators to meet the challenges of the digital age. In this context, Digital Teaching Competence (DTC) is vital, allowing educators to use technology effectively while fostering interactive and dynamic learning environments that support student success. This section outlines the key theoretical concepts, dimensions, evaluation models, and self-assessment tools like DigCompEdu Check-in, emphasizing DTC’s transformative role in higher education and its importance for professional development.

***Digital Teaching Competence (DTC)***

Digital Teaching Competence (DTC) in higher education refers to the set of knowledge, skills, and abilities necessary for the effective use of digital media in an educational context. ^20^ This competence enables educators to achieve pedagogical objectives through the use of technologies, making the design, implementation, and execution of training initiatives aimed at incorporating digital tools an essential professional requirement. ^21^ Various studies highlight that DTC is an evolving skill that advances in parallel with rapid technological developments, requiring continuous updating by educators. ^22^^,^^23^

***Environmental Factors in Technological Integration***

The integration of technology in education does not solely depend on the availability of technological tools but also on various environmental factors, such as institutional support, educational policies, and available technological infrastructure. ^24^ In this regard, DTC should be conceived as a dynamic competence linked not only to technological advances but also to educators’ ability to adapt to changing educational contexts. ^25^ When applied through interactive pedagogical tools, DTC enhances learning and contributes to the creation of more meaningful educational environments. ^26^

***Dimensions and Evaluation Models of DTC***

Within the framework of DTC, different dimensions are identified, categorized by models and standards established for its evaluation. The International Society for Technology in Education (ISTE) and the National Institute of Educational Technologies and Teacher Education (INTEF) propose five areas of digital competences for educators: information and information literacy, communication and collaboration, digital content creation, security, and problem-solving. ^27^^–^^29^ These areas are organized into competence levels, from basic (A1, A2) to intermediate (B1, B2) and advanced (C1, C2), depending on the educator’s proficiency in using digital technologies. ^30^^,^^31^

***Transformative Importance of DTC in Higher Education***

In the context of higher education, several studies have demonstrated that DTC has a transformative impact. Educators not only need to develop their own digital skills but also foster the necessary competencies in students to adapt to the digital world. ^31^^,^^32^ These studies reveal that DTC is crucial in preparing teachers and students for contemporary technological challenges. Moreover, its relevance is reinforced in the context of the United Nations’ Sustainable Development Goals (SDGs), where digital competences contribute to advancing equality and social progress.

***Operational Definition of DTC***

DTC is operationally defined as the ability of educators to develop operational skills with the use of technological devices, facilitating access to information and digital resources. This competence has evolved into a flexible and critical tool that adapts to social and educational realities and continuously evolves. Its integration into the educational field has allowed for the development of frameworks and self-assessment tools that measure the degree of DTC appropriation, both in educators and educational managers. ^31^ These tools are aligned with educational policies and strategies proposed by organizations such as UNESCO and the European Union.

***Importance of Digital Teaching Competence (DTC) and Evaluation Tools***

The literature review highlights that the importance of Digital Teaching Competence (DTC) has been widely recognized through the development of frameworks and self-assessment instruments designed to measure the degree of DTC appropriation by teachers and educational managers, as well as the integration and use of Information and Communication Technologies (ICT). ^33^^,^^34^ These frameworks and instruments align closely with educational policies and strategies proposed by Digitally Competent Educational Organizations, the European Framework for Digital Competence of Educators (DigCompEdu), the Mentoring Technology-Enhanced Pedagogy (MENTEP) project, and the United Nations Educational, Scientific and Cultural Organization (UNESCO). ^24^

***International Projects to Promote DTC***

UNESCO’s Mentoring Technology-Enhanced Pedagogy (MENTEP) project aims to incorporate the technical model of ICT into the pedagogical environment to enhance teaching through technology, ^24^^,^^35^ particularly through the use of Massive Open Online Courses (MOOCs). ^36^ This project also provides teachers with the opportunity to self-assess their digital competences and identify areas for improvement through the standardized TET-SAT test. ^24^ This initiative emphasizes the importance of self-assessment in acquiring and strengthening digital competences in educational settings.

***The European Framework for Digital Competence of Educators (DigCompEdu)***

The European Framework for Digital Competence of Educators (DigCompEdu) provides a solid reference for the development of digital competences at all educational levels. ^37^^,^^38^ Based on scientific foundations, DigCompEdu helps identify needs, deepen knowledge, expand skills, and promote professional development. ^39^ This framework covers six competence areas: professional engagement, digital resources, teaching and learning, assessment and feedback, student empowerment, and fostering students’ digital competence. ^40^^,^^41^ These areas stand out for their comprehensive approach to DTC development, adapting to various educational contexts and levels of proficiency.

## Methods

To explore the proposed objectives, a descriptive correlational study was designed, supported by a quantitative approach and a non-experimental design structure. The methodological choice was neither random nor incidental but was deliberately aligned with the overall goals of the research. The focus was on delving into the essential core of the studied phenomenon and the intricate relationships between predetermined variables. ^42^ It is noteworthy that the strategy adopted is based on a strictly observational model, excluding any form of direct intervention or manipulation of the subject matter.

Within this framework, the following key elements are established:

• Critical phases in the research process were meticulously identified. These stages include the recruitment period for participants scheduled for the year 2022, specific moments for exposing the study subjects to the variables of interest, carefully structured follow-up phases, and predetermined timeframes for the empirical data collection.
• Proactive measures were taken to identify and mitigate potential sources of bias. For this, statistical methods such as random probability sampling and weighted tests were applied. These techniques were specifically adapted to the peculiarities and demands of the research with the aim of preserving objectivity and impartiality in the results.
• The selection of the number of participants was based on advanced statistical algorithms. These considered both the analytical power and the estimated effect size of the variables in question. This dual approach not only maximized the capability to detect significant interactions or impacts but also minimized the risks of erroneous inferences. In the event of missing data, consolidated statistical methodologies are applied. Initially, the nature and extent of the missing data were assessed. Subsequently, data imputation techniques were opted for, or alternatively, analyses based on complete records were carried out. This procedure ensured the integrity and reliability of the data and its subsequent interpretations.

### Ethical considerations

In accordance with the ethical imperatives governing academic research, a meticulous procedure for obtaining informed consent from participants was diligently executed prior to the administration of the research instrument. This is a critical facet in the realm of scientific inquiry, designed to ensure that participants are not only fully aware of the study’s overarching aim and methodology but also of any prospective risks and benefits that may arise from their involvement.

To facilitate a comprehensive understanding of the study’s parameters, informed consent documents were articulated in a lucid and accessible language, deliberately avoiding any complex technical terminology that could potentially obfuscate participants’ comprehension of the study’s scope and implications. This approach was adopted to reinforce the principle of voluntariness, emphasizing that participants were free to either abstain from or withdraw from the study at any point, without suffering any negative repercussions.

Simultaneously, at the same time, strict protocols were established to safeguard the confidentiality and privacy of the data collected from the participants. Detailed explanations were provided regarding the mechanisms to protect the identity and personal data of the participants. Once any pending questions or concerns were addressed, participants were invited to officially register their consent. This was achieved through the signing of the informed consent document, before proceeding to administer the questionnaire through Google Forms.

The authorization for the execution of the current research was granted by the Institutional Ethics Committee, and the funding was facilitated by the Honorable University Council of the Technical University of Manabí. This support was institutionalized through the issuance of resolution RHCU.UTM-No.259-SO-10-2022, dated January 10, 2022, thus preceding the data collection phase.

### Participants and sampling

The study population comprised the entire faculty body of the Technical University of Manabí, totaling 992 academic professionals (N = 992). A non-probabilistic, purposive, and voluntary sampling method was employed, resulting in an estimated sample of 277 faculty members (n ≈ 277). The sample size was determined using the finite population sampling formula proposed by Hernández *et al.*, ^43^ which takes into account the population size, confidence level, estimated proportion, and acceptable margin of error. This approach ensured adequate capacity to detect significant interactions while guaranteeing that the sample size was representative of the finite population under study. Although the voluntary nature of the sampling may limit the representativeness of the sample relative to the entire population, this method was deemed the most appropriate given resource constraints and the specific objectives of the research.

### Data collection instrument

The DigCompEdu Check-in was employed as the data collection instrument for this study, originally developed by Ghomi and Redecker ^17^ and published by the Joint Research Center. This tool has been widely used to assess digital teaching competencies in various educational contexts. While the instrument was initially adapted to the Spanish context by Cabero-Almenara and Palacios-Rodríguez, ^44^ adjustments were necessary to ensure its relevance and applicability to the Ecuadorian educational setting. The adaptation process considered the distinct cultural, demographic, socio-educational, and economic factors present in Ecuador, which differ significantly from those of the European context where the tool was first applied.

The adaptation of the instrument to the Ecuadorian context was carried out with theoretical and methodological rigor, ensuring that it accurately reflected the realities of local educational systems. Ecuador, like many other Latin American countries, faces unique challenges related to access to technology, infrastructure limitations, and varying levels of digital literacy across different regions and populations. These factors were considered when adapting the survey, ensuring that the instrument captured the specific needs and competencies relevant to Ecuadorian educators.

Additionally, the socio-economic disparities present in Ecuador necessitated a more inclusive approach to assessing digital competencies, as educators may have varying levels of access to digital resources. The adaptation involved reviewing the language, examples, and scenarios used in the questionnaire to reflect the local educational environment and the realities of teaching in both urban and rural settings. Moreover, this adjustment aligned with recommendations from authors such as Martínez-Bravo *et al.*, ^45^ who emphasize the importance of contextualizing digital competency frameworks to account for regional educational disparities and technological constraints.

The final version of the instrument retained its original structure, comprising 22 items categorized into six domains: Professional Commitment, Digital Resources, Digital Pedagogy, Assessment and Feedback, Empowering Students, and Facilitating Students’ Digital Competence. The responses were measured using a five-point Likert scale, ranging from “strongly disagree” to “strongly agree,” and supplemented with demographic variables such as gender and age. The items were coded alphanumerically, with the first letter representing the specific competency domain (e.g., “C” for Professional Commitment and “R” for Digital Resources).

The survey was distributed via Google Forms, ensuring efficient data collection and anonymity of responses, thus adhering to both ethical and logistical requirements. This online distribution method was particularly relevant in the Ecuadorian context, where accessibility to digital tools can vary, but the widespread availability of internet connections in academic institutions allowed for broad participation.

### Data analysis

Following the data collection via surveys, an inferential analysis was conducted with the aim of elucidating the self-perception that educators have regarding their digital competencies. To eliminate ambiguities in the categorization of these competencies, a secondary alphabetical character was assigned to each group when two groups shared the same initial letter. Thus, “Evaluation and Feedback” was coded as “EfV,” while “Empowerment” was labeled as “EP.”

During the variable construction phase, a composite index (CALIF) was calculated for each participant. This was achieved by summing the corresponding values of the responses for the items linked to each competency domain. The composite index ranged from 0 to 88 points. Once these scores were obtained, the individuals’ competency levels were categorized according to the grading scheme outlined in Table 1.

**Table 1.** Classification and scoring system of the "DigCompEdu Check-In" competence level.

Level of competence	Score (out of 88 points)
Novice (A1)	<20
Explorer (A2)	20-33
Integrator (B1)	34-49
Expert (B2)	50-65
Leader (C1)	66-80
Pioneer (C2)	>80

Source: Ref. 46.

For each area of competence, a variable was generated with the sum of the scores of the items that constituted the area of competence, and together with each numerical variable, a categorical variable was conceived as suggested by Ref. 46, as specified below:

• **COMP_C (Commitment):** Based on responses in “Professional Commitment,” with scores ranging from 0 to 16 points.
• **PEDAGO_C (Digital Pedagogy):** Based on responses in “Digital Pedagogy,” with scores ranging from 0 to 16 points.
• **RECDIGC (Digital Resources):** Based on responses in “Digital Resources,” with scores ranging from 0 to 12 points.
• **EMPODERAC (Empowerment):** Based on responses in “Empowering Students,” with scores ranging from 0 to 12 points.
• **EVALUAYRC (Evaluation and Feedback):** Based on responses in “Evaluation and Feedback,” with scores ranging from 0 to 12 points.
• **FACILITACOC (Facilitating Students’ Digital Competence):** Based on responses in “Facilitating Students’ Digital Competence,” with scores ranging from 0 to 20 points.

This structured approach facilitated a comprehensive analysis of the various dimensions of digital competencies among educators, ensuring that each competency area was accurately represented and analyzed.

The reliability of the DigCompEdu Check-in instrument was evaluated using Cronbach’s Alpha to assess both general and internal consistency. The analysis was conducted with SPSS-21 software, yielding a Cronbach’s Alpha of 0.949 across the 22 items, which signifies a high level of reliability (see Table 2).

**Table 2.** Reliability statistics.

Reliability statistics	Value	Number of elements
Cronbach’s Alpha	0.949	22

A reliability test using Cronbach’s alpha coefficient was performed to assess the internal consistency of the 22 survey items, providing an indication of how well the instrument consistently measures the same underlying construct. A higher Cronbach’s alpha value signifies greater reliability, while a lower value suggests potential inconsistencies. The analysis, conducted with statistical software like SPSS-21, confirmed the robustness and stability of the instrument, ensuring that the items consistently reflect the digital competencies being assessed. The results, shown in Table 3, demonstrate the instrument’s overall reliability, reinforcing the validity of the findings.

**Table 3.** Cronbach's reliability test results.

Area	Competence	Scale mean if the item has been deleted	Scale variance if the item has been deleted	Correlation total corrected items	Cronbach's alpha if the item has been deleted
Professional Engagement	Organizational Communication	52.5343	129.018	0.500	0.949
	Professional Collaboration	52.8448	128.885	0.611	0.948
	Reflective Practice	52.5812	127.708	0.627	0.947
	Digital Training	51.9458	124.283	0.654	0.947
Digital Resources	Selection	52.5668	128.609	0.596	0.948
	Creation and Modification	52.5307	129.388	0.623	0.948
	Administration, Sharing, and Protection	52.3827	126.686	0.607	0.948
Digital Pedagogy	Teaching	52.4585	125.698	0.694	0.947
	Guidance	52.1913	127.510	0.703	0.946
	Collaborative Learning	52.2888	128.699	0.640	0.947
	Self-directed Learning	52.2166	129.272	0.669	0.947
Evaluation and Feedback	Evaluation Strategies	52.2635	128.166	0.702	0.947
	Analysis of Evidence and Tests	52.3863	126.252	0.716	0.946
	Feedback and Planning	52.6643	127.840	0.723	0.946
Empowering Students	Accessibility and Inclusion	52.1047	128.029	0.642	0.947
	Differentiation and Personalization	52.4693	126.235	0.677	0.947
	Active Student Participation	52.3899	125.492	0.751	0.946
Facilitating Students' Digital Competence	Information and Media Literacy	52.5235	127.316	0.689	0.947
	Digital Communication and Collaboration	52.4513	127.401	0.700	0.947
	Creation of Digital Content	52.2491	127.115	0.718	0.946
	Responsible Use and Well-being	52.4801	126.555	0.735	0.946
	Digital Problem Solving	52.4296	127.688	0.653	0.947

Table 3 presents the results of Cronbach’s alpha reliability assessment across several key competency domains, including Professional Engagement, Digital Resources, Digital Pedagogy, Evaluation and Feedback, Empowering Students, and Facilitating Students’ Digital Competence. The analysis reveals high reliability across all competencies, with Cronbach’s alpha values ranging from 0.946 to 0.949, surpassing the commonly accepted threshold of 0.7, which indicates substantial internal consistency. These results suggest that the instrument consistently measures the intended competencies across the various domains.

Further analysis of the scale mean and variance in the event of item deletion shows minimal variation, with the scale mean averaging around 52.5 and variance values ranging between the mid-120s and 130. This consistency across competencies implies a balanced scale. Additionally, correlations between individual competencies and the corrected total score reveal positive linear relationships, with coefficients ranging from 0.500 in Organizational Communication (Professional Engagement) to 0.751 in Active Student Participation (Empowering Students). This indicates that an increase in individual competency scores correlates with a rise in the overall score. Lastly, evaluating Cronbach’s alpha when specific items are removed suggests that the exclusion of any single competency would have a negligible impact on the overall reliability of the instrument, further reinforcing its robustness.

## Results

In this section, the results of the study are presented, building on the work of Moreira-Choez *et al.*, ^70^ provide a detailed analysis of participants’ self-evaluations of their digital teaching competencies. Using descriptive statistics, the data were interpreted to reveal trends, frequencies, and averages, helping to identify key insights regarding faculty members’ proficiency in various digital competency areas. The analysis utilized a five-level Likert scale, ranging from “strongly disagree” to “strongly agree,” allowing for a nuanced interpretation of the responses. This categorization highlighted general trends and specific areas where digital competency training may be needed, offering a foundation for future discussions and recommendations aimed at improving digital teaching practices within the institution.

**Table 4.** Results of the descriptive analysis on the areas of digital competence of professors (n=277).

	Categories					
Competence	Novice	Explorer	Integrator	Expert	Leader	Pioneer
PROFESSIONAL COMMITMENT	3.25	16.97	36.46	38.99	3.61	0.72
RECOGNIZES EVALUATES AND EMPOWERS	2.53	13.00	39.35	35.38	9.03	0.72
DIGITAL PEDAGOGY	0.36	9.39	40.07	39.71	7.94	2.53
EVALUATES AND PROVIDES FEEDBACK	1.44	9.03	46.21	32.13	7.58	3.61
EMPOWERS STUDENTS	2.53	8.30	32.85	42.60	9.03	4.69
FACILITATES COMPETENCES	2.53	5.05	48.74	36.82	3.61	3.25

Source: data provided by the respondents Moreira-Choez *et al.*, ^70^ processed with SPSS-21 statistical software.

Table 4 presents the distribution of participants (n=277) across different competence levels for six key areas of digital competence. The results provide a comprehensive overview of how professors self-assess their proficiency in these domains, highlighting areas of strength and potential improvement.

In the area of Professional Commitment, the majority of professors are categorized as experts (38.99%) and integrators (36.46%). These findings indicate a significant level of engagement in professional development and digital competence. However, only a small proportion of respondents are classified as pioneers (0.72%) or novices (3.25%), suggesting a lower presence of both extreme levels of competence. Intermediate categories, such as explorers and leaders, account for 16.97% and 3.61% respectively.

The Recognizes, Evaluates, and Empowers competency shows a similar trend, with the integrator category representing the largest group (39.35%), followed closely by experts (35.38%). Novices and pioneers are underrepresented, with values of 2.53% and 0.72% respectively, while explorers (13.00%) and leaders (9.03%) occupy intermediate levels. This distribution reflects an overall positive self-perception among the majority of respondents, though it indicates room for growth in leadership and pioneering roles.

In the Digital Pedagogy domain, integrators (40.07%) and experts (39.71%) dominate the responses, demonstrating high levels of competence in the application of digital tools in teaching practices. Explorers account for 9.39%, while leaders (7.94%) and pioneers (2.53%) remain in the minority. Only 0.36% of respondents identify as novices, indicating a general familiarity with digital pedagogical tools across the sample.

For the Evaluates and Provides Feedback competence, the largest group consists of integrators (46.21%), followed by experts (32.13%). Explorers (9.03%) and leaders (7.58%) show a moderate presence, whereas pioneers (3.68%) and novices (1.44%) constitute the smallest categories. This suggests that most professors are proficient in using digital tools for evaluation and feedback, with fewer at the novice or pioneer levels.

Regarding Empowers Students, 42.60% of the participants identify as experts, and 32.85% as integrators. The remaining categories include leaders (9.03%), explorers (8.30%), pioneers (4.69%), and novices (2.53%), illustrating that the majority of professors feel confident in empowering students through digital means, though there is still a notable proportion in lower competence levels.

Finally, the Facilitates Competences competence is predominantly represented by integrators (48.74%) and experts (36.82%). The explorer category accounts for 5.05%, while leaders (3.61%), pioneers (3.25%), and novices (2.53%) remain less frequent. This distribution indicates that a considerable number of professors perceive themselves as proficient in facilitating digital competencies in their educational practices.

Table 5 presents the analysis of variance, which examines the sources of variation, the degrees of freedom, and the sum of squares for each numerical variable investigated. Analysis of variance (ANOVA) is a statistical technique used to determine the significance of differences between groups or categories.

**Table 5.** Summary of variance analyses.

Source of variation	gl	Commitment	DigResources	Digital pedagogy	Empowers	Evaluatesandpro	Facilitatesco
Sex	1	0.194	11.978	3.956	0.561	3.168	14.771
Academic profile	3	13.812	6.320	4.432	5.814	0.963	10.764
Age	3	5.725	7.215	30.762	20.350	16.314	59.216
Sex x academic profile	3	42.867	23.227	**46.227**	17.368	11.432	43.846
Sex x Age	3	31.892	9.857	15.598	6.605	6.975	27.227
Academic profile x Age	5	11.394	6.288	13.966	17.036	8.279	32.639
Sex x academic profile x Age	2	8.429	11.755	8.799	3.864	2.409	2.676
Error	257	1635.739	857.105	1386.356	1039.333	880.300	2313.342
**Total corrected**	277	1805.726	954.671	1500.686	1104.801	932.108	2533.199

Source: Data provided by respondents Moreira-Choez *et al.*, ^70^ processed with SPSS-21 statistical software.

When examining the sources of variation such as sex, academic profile, and age, as well as their interactions, significant differences are observed in the “pedagogy variable” due to the interaction effect between sex and academic profile. This suggests that the relationship between sex and academic profile influences the variability in pedagogical competence.

Furthermore, the relationship between academic profile and age accounts for approximately 31.892% of the variability in professional commitment. This indicates that the combination of academic profile and age significantly contributes to variations in the level of professional commitment.

Similarly, the confluence of sex, academic profile, and age has a notable impact, accounting for 11.755% of the variability in digital resources. This suggests that the interaction among these factors plays a significant role in determining the level of digital resource competency.

Table 6 provides a summary of the Chi-Square test results, which examines the independence of each factor against all categorical variables in each competence area. The “P value” column indicates the significance of each test, with a significance criterion of α=0.05. If the test is not significant, it suggests that the variables are independent. However, for example, in the case of the “Evaluates and provides feedback” variable, a significant relationship with sex is found.

**Table 6.** Chi-square test for the factors of sex, age and academic education level.

Factor	Levels	Chi squared	Gl	P value
**Sex**	Professional commitment	3.767 ^a^	5	0.583
	Digital pedagogy	4.983 ^a^	5	0.418
	Recognizes, evaluates	6.142 ^a^	5	0.293
	Evaluates and provides feedback	15.543 ^a^	5	0.008
	Empowers students	7.204 ^a^	5	0.206
	Facilitates competences	1.903 ^a^	5	0.862
**Age**	Professional commitment	29.113 ^a^	15	0.016
	Digital pedagogy	32.688 ^a^	15	0.005
	Recognizes, evaluates	36.243 ^a^	15	0.002
	Evaluates and provides feedback	24.878 ^a^	15	0.052
	Empowers students	15.910 ^a^	15	0.388
	Facilitates competences	42.415 ^a^	15	0.0001
**Academic profile**	Professional commitment	15.540 ^a^	15	0.413
	Digital pedagogy	24.410 ^a^	15	0.058
	Recognizes, evaluates	9.339 ^a^	15	0.859
	Evaluates and provides feedback	20.957 ^a^	15	0.138
	Empowers students	28.461 ^a^	15	0.019
	Facilitates competences	16.066 ^a^	15	0.378

Source: Data provided by respondents, processed with SPSS-21 statistical software.

Regarding age, independence is observed in the “Evaluates and provides feedback” and “Empowers students” variables, suggesting that age does not significantly affect these competences. However, in the other variables, a significant relationship is found between age and the rest of the competences, indicating a non-independent effect of age on those variables.

For the academic profile, independence is found in relation to age, except in the “Empowers students” variable. This implies that the academic profile has a significant relationship with age in most competences, except in the case of empowering students.

These findings highlight the complex interplay of factors and their interactions in influencing the variations observed in different competence areas. Understanding these relationships is essential for tailoring educational interventions and designing targeted strategies to enhance specific competences based on sex, academic profile, age, and their combinations.

**Figure 1.**
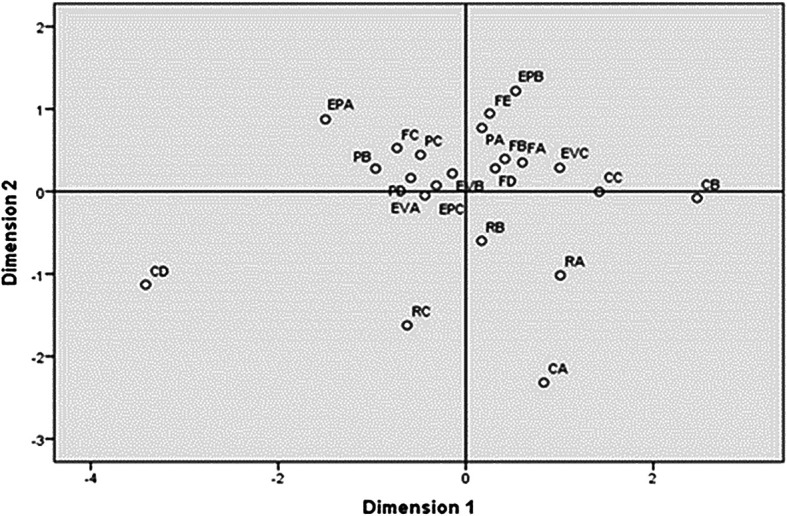
Scaling of competences. Source: Data provided by respondents, processed with SPSS-21 statistical software.

Within the possibilities of multidimensional scaling with the application of the multivariate technique, stress is presented as a measure of goodness of fit, whose indicator was ‘weak to poor’ with a value of 0.1828, which is reflected in the graph represented by a regression in Figure 1, where the degree of coupling of the data can be observed. However, in the Stress value, it was visualized that there was closeness between the items of the ‘Empowers’, ‘Evaluates’, ‘Facilitates Digital Competence’ competence areas. Therefore, the items of these areas of competence formed two compact groups of items, sharing items between one group and the other, but indicating that in general these items formed a construct.

As it can be observed **,** it was verified that Professional Commitment and Digital Resources were left out of this group. In this way, it was observed that the most dispersed items were those of Professional Commitment. On the other hand, only “Reflective Practice” and “ Professional Collaboration “ were relatively close to each other, but very far from “Digital Training” and “Organizational Communication”. Likewise, the “Digital Resources” items were found to be close to each other. Consequently, it could be considered that the items of these two areas formed another construct.

Next, a two-stage factor analysis was performed.

The results of the factor analysis with the maximum likelihood method and the “Oblimin” oblique rotation method option showed a KMO value of 0.961 and Bartlett’s test of sphericity was significant, indicating that the application of the factor analysis was adequate.

The lower part of Table 7 shows the characteristic values of the first two factors, and it was observed that with the first two factors a cumulative explained variance of 56.58% was obtained which was considered acceptable, suggesting a two-factor model. The first factor was made up of the competences that were mainly aimed at the achievement of empowerment by students of digital tools to apply them to the teaching-learning process.

**Table 7.** Factor analysis of items

Area/Competence	Factor	
	1	2
Facilitating Students' Digital Competence/Digital Communication and Collaboration	0.859	
Facilitating Students' Digital Competence/Responsible Use and Well-being	0.809	
Empowering Students/Differentiation and Personalization	0.800	
Facilitating Students' Digital Competence/Digital Problem Solving	0.788	
Facilitating Students' Digital Competence/Creation of Digital Content	0.787	
Facilitating Students' Digital Competence/Information and Media Literacy	0.702	
Evaluation and Feedback/Feedback and Planning	0.659	
Evaluation and Feedback/Analysis of Evidence and Tests	0.631	
Digital Pedagogy/Collaborative Learning	0.597	
Empowering Students/Accessibility and Inclusion	0.595	
Empowering Students/Active Student Participation	0.590	
Digital Pedagogy/Self-directed Learning	0.472	
Digital Pedagogy/Guidance	0.461	
Digital Pedagogy/Teaching	0.393	0.373
Professional Engagement/Digital Training		0.783
Digital Resources/Selection		0.678
Digital Resources/Creation and Modification		0.640
Professional Engagement/Organizational Communication		0.633
Professional Engagement/Reflective Practice		0.442
Evaluation and Feedback/Evaluation Strategies	0.368	0.409
Digital Resources/Administration, Sharing, and Protection		0.373
Auto value	10.862	1.285
% Variance explained	49.374	5.840
% of cumulative variance	49.374	55.214

Source: Data provided by respondents, processed with SPSS-21 statistical software.

The second factor generated a construct that described the professors’ personal attitudes toward their training and use of digital tools. That is, construct one referred to the entire pedagogical structure, and construct two to the professors’ personal training and use of it.

Similarly, Table 7 presents the factors and the loadings of each of the items within the factors. Thus, the rotation analysis made it possible to achieve what the literature suggests as a good result in factor analysis, in other words, that each factor is made up of variables with high values and some variables with values close to zero, and that each variable belongs to only one of the factors.

Therefore, variables with small values in the factors were omitted to avoid duplicities, taking as a criterion for the elimination of values lower than 0.35. For this reason, only two items could not be fully discriminated against, which were Professional Collaboration and Evaluation Strategies. However, Professional Collaboration was more important in factor 1, while Evaluation Strategies was more important in factor 2. Finally, it was observed that the item Digital Training was not part of any of the factors to obtain the scores.

**Figure 2.**
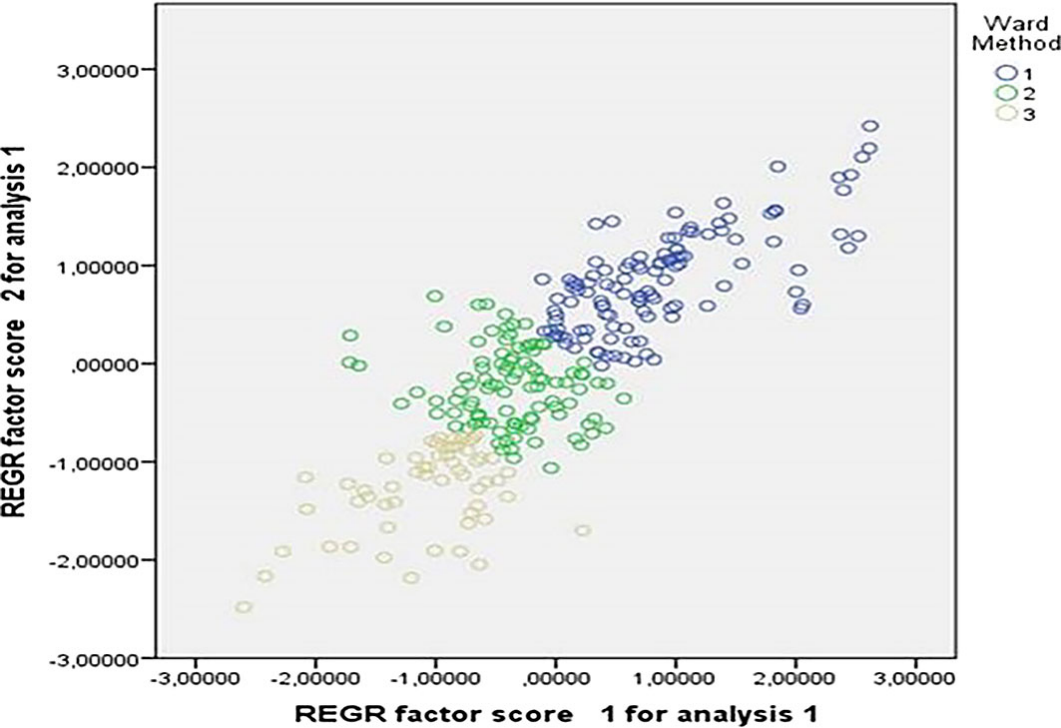
Assisted clustering analysis of the constructs. Source: Data provided by respondents, processed with SPSS-21 statistical software.

It is clear that through the assisted clustering analysis, three groups were identified. These are shown in Figure 2.

In this analysis, it was found that the grouping of participants was strongly influenced by factor 1, which pertained to the pedagogical aspect. Group 1 comprised professors who exhibited higher ratings on construct 1, indicating a strong emphasis on pedagogy. Group 2 represented an intermediate category, characterized by a balance between the two constructs. On the other hand, Group 3 consisted of professors whose ratings on construct 2 prevailed.

These groupings suggest that participants varied in their emphasis on different aspects of teaching and learning. Group 1 highlighted a strong focus on pedagogical approaches, while Group 3 demonstrated a greater emphasis on the second construct, which may be related to other dimensions or competences.

Understanding these groupings provides insights into the diverse perspectives and preferences of educators in relation to teaching practices and competences. It enables researchers and educators to identify specific areas where professional development and support can be targeted to enhance teaching effectiveness and promote a well-rounded approach to education.

To describe more precisely the constitution of the groups, the means corresponding to each of the variables that calculated the score in each area of competence were generated, the results of which are shown in Table 8.

**Table 8.** Means and standard deviation of the groups formed.

Variable	G1 (n=117)		G2 (n=101)		G3(n=59)	
	Mean	Standard deviation	Mean	Standard deviation	Mean	Standard deviation
COMMITMENT	11.85	1.428	9.0792	1.56002	6.509	1.569
DIGRESOURCES	8.69	1.296	6.8119	1.14641	5.034	1.144
DIGPEDAGOGY	12.35	1.698	9.8119	1.42627	7.797	1.200
EMPOWER ESTUD	9.29	1.327	7.1683	1.49713	5.661	1.360
FACILITATESCO	14.86	2.297	11.1782	1.83519	9.509	1.995
EVALUATES AND PROVIDES FEEDBACK	8.85	1.538	6.7426	1.00651	5.610	1.145

Source: Data provided by respondents, processed with SPSS-21 statistical software.

To provide a more detailed description of the group composition, the means for each variable that contributed to the calculation of the score in each competence area were computed. The results of this analysis are presented in Table 8.

Table 8 displays the mean values for each variable within each competence area. These means offer a comprehensive understanding of the specific aspects or dimensions that contribute to the overall score in each competence area. By examining the means, researchers can identify the relative strengths and weaknesses of the participants in different areas of competence.

Analyzing the means enables a more nuanced interpretation of the group composition and allows for a deeper exploration of the participants’ competences. It provides valuable insights into the specific areas where educators excel or require further development.

By considering these mean values, researchers and educators can design targeted interventions and strategies to enhance specific competences and address any areas of improvement identified. This information contributes to the overall goal of fostering professional growth and enhancing the quality of teaching and learning experiences.

## Discussion

Upon examining the results, it is evident that the Integrator and Expert categories consistently present higher percentages in various competencies, such as PROFESSIONAL COMMITMENT, RECOGNIZE, EVALUATE AND EMPOWER, DIGITAL PEDAGOGY, EVALUATE AND PROVIDE FEEDBACK, EMPOWER STUDENTS, and FACILITATE COMPETENCIES. This indicates that teachers who perceive themselves as integrators and experts demonstrate a higher level of Digital Teaching Competence compared to explorers and novices. Additionally, leaders and pioneers, albeit fewer in number, show greater knowledge and abilities in these competencies.

These findings align with studies conducted by Espino-Díaz *et al.*, ^47^ and Hämäläinen *et al*. ^48^ which similarly highlight a pattern of behavior in competencies leaning towards a low to medium competence level. The study by Peled ^49^ also supports this idea, suggesting that there is a positive correlation between teachers’ self-perception as experts or integrators and their level of Digital Teaching Competence.

In terms of demographic factors, Table 5 shows that sex and academic profile exhibit the highest values on the digital pedagogy variable. This suggests that, regardless of sex, educational teaching techniques are applied without gender bias, contributing to the development of Digital Teaching Competence. Furthermore, the lower values imply that sex is not a significant factor in acquiring commitments or fulfilling professional responsibilities in teaching.

These findings align with the discoveries of Guillén-Gámez *et al.*, ^50^ who emphasize the limited differences between sex and age in terms of self-perception of digital competencies. This corroborates that sex does not have a significant impact on learning and teaching abilities related to the use and management of digital tools. It also suggests the need to plan and implement strategies for integrating digital competencies into teacher training programs.

Regarding age, the percentages indicate that it is not perceived as a limitation for acquiring or fulfilling commitments related to the appropriation of digital resources. This finding aligns with Suárez and Colmenero, ^51^ where no statistically significant association was found between age and competence level, indicating that there are no significant differences among different age groups. This result is also backed by the study of Gudmundsdottir and Hatlevik, ^52^ suggesting that digital competence is not determined by age, but rather by attitude and training.

Table 6 examines the relationship between factors, including sex, age, and academic profile. It indicates that sex is independent of the variables, except for “Evaluate and provide feedback,” suggesting that sex influences the search for possibilities to identify and verify knowledge. Also, the “Evaluate and provide feedback” and “Empower students” variables show the lowest values, indicating that age does not determine processes involving communication with students.

Regarding the academic profile, the “Empower students” variable obtains the highest score, indicating that teachers strive to provide students with the necessary support and information to guide their development. Although some teachers possess basic or intermediate digital skills, it is essential that they empower their students through adequate training. This aligns with the study conducted by Colás-Bravo *et al*., ^53^ emphasizing the need for teachers to contribute to developing critical, reflective, creative, and innovative thinking in students through proper training. Aidoo *et al.* ^54^ study supports this idea, suggesting that teachers need to have a deeper understanding of how to use digital tools to empower students and foster their autonomous learning.

The correlation analysis presented in Figure 1 highlights the interrelationship between items related to competencies such as “Empower”, “Evaluate”, and “Facilitate Digital Competence.” This suggests that as teachers set out to empower students, they contribute their own competencies to improve students’ skills, thus demonstrating their commitment to professional performance and teaching praxis.

However, it is important to note that Professional Commitment and Digital Resources were not included in this construct, indicating that not all teachers share the same attitude towards student training, possibly due to resource limitations. Another construct formed by “Digital Training” and “Organizational Communication” suggests the existence of divided positions regarding empowerment practices.

Findings from List ^55^ support this idea, as they indicate that the management of digital identities in the educational context, specifically in the dimensions of communication and collaboration, is limited among teachers. This suggests that there is a need for further development of digital competencies among educators. Similarly, the study of Benitt *et al.* ^56^ concludes that teachers need more comprehensive training in digital competencies to effectively utilize technologies in teaching.

Table 7 highlights two significant factors: Professional Collaboration and Evaluation Strategies, both of which play crucial roles in integrating digital competencies in education. Professional Collaboration, identified as a key component of the first factor, underscores the importance of adapting virtual environments to foster collaborative learning. This competency not only facilitates the application of strategies for enhancing student engagement but also contributes to creating more inclusive and participatory learning environments, as supported by Kempe and Grönlund, ^57^ who emphasize the need for collaborative digital practices in contemporary educational contexts. According to Majid and Ali, ^58^ digital collaboration among teachers is essential for building learning communities that promote shared knowledge and innovation. Therefore, promoting teachers’ participation in professional development programs that focus on collaboration in digital environments becomes critical to advancing pedagogical practices and responding to the evolving demands of online education. ^59^^–^^61^

The second factor, Evaluation Strategies, highlights the essential role of assessment in determining student learning outcomes and identifying areas for pedagogical improvement. This aligns with findings by Amhag *et al.*, ^62^ who assert that effective digital evaluation tools are indispensable for modern education, allowing teachers to track performance and adjust instruction accordingly. As Sillat *et al.* ^63^ suggest, digital competence training programs must incorporate emerging methodologies for assessment, such as formative and summative evaluations using technological platforms. Additionally, Kaswan *et al*. ^64^ argue that leveraging digital tools in assessment processes enables more personalized feedback and continuous improvement in teaching methods. Evaluation strategies are, therefore, pivotal for ensuring that pedagogical practices evolve alongside technological advancements, equipping educators with the ability to organize, track, and improve learning outcomes in virtual environments. By adopting these methods, educational institutions can enhance the quality of learning and better prepare students for future challenges in a digitized society. ^65^^–^^67^

Figure 2 identifies three distinct groups of teachers. The first group (G1) emphasizes the importance of grades in pedagogical processes, while the second group (G2) lacks a clear definition. The third group (G3) expresses limited contributions to the topic. These findings are consistent with the conclusions drawn by Gil-Jaurena and Domínguez, ^41^ who recommend incorporating more profound educational technology content into training programs to improve educational quality and enable appropriate teaching praxis for the digital age. Moreover, the findings of the study by Rapanta *et al* ^68^ support this recommendation, suggesting that a better understanding of educational technology can help teachers adapt their practices to students’ needs and expectations in the digital environment.

Finally, Table 8 presents the mean values and standard deviations of the formed groups. It reveals that Group 1 (G1) shows the lowest proportional effect on the “DIG RESOURCES” variable, indicating that without adequate digital resources, teachers face limitations in providing quality education. On the other hand, Group 1 (G1) shows the highest weighted average on the “FACILITATESCO” variable, suggesting that the ability to facilitate required competencies largely depends on the interaction between teachers and their students. In Group 3 (G3), the “DIGRESOURCES” variable receives the lowest scale, underlining the importance of having digital resources to promote skills for effective use of digital environments. These findings align with the study conducted by Pozo-Sánchez *et al*., ^69^ which associates higher digital competence scores with dimensions related to information and communication literacy and collaboration.

The study provides a comprehensive view of how teachers perceive themselves in terms of digital competence and how this perception relates to demographic variables such as gender, age, and academic profile. However, certain limitations and ambiguous areas suggest the need for further and more detailed research in the future.

## Conclusions

The descriptive analysis revealed valuable insights into the self-perception of digital teaching competence among professors at the Technical University of Manabí (UTM). The majority of participants demonstrated a tendency towards the “Integrator” and “Expert” categories, with competencies such as “Facilitating Competences,” “Evaluating and Providing Feedback,” and “Digital Pedagogy” being the most frequently highlighted. Furthermore, the study found significant relationships between certain demographic variables and professors’ digital competence. Specifically, age, sex, and academic profile were shown to influence competencies in the “Digital Pedagogy” domain, although the relationship between sex and academic profile appeared inconsistent. Most variables displayed independence except for “Evaluates and Provides Feedback,” which demonstrated statistical significance.

### Limitations

One of the primary limitations of this study is the lack of random sampling, which restricted the creation of a balanced and homogeneous sample. The non-probabilistic nature of the sample may have introduced bias, potentially affecting the generalizability of the findings to the broader population of university professors. Additionally, the voluntary participation of professors may have resulted in the underrepresentation of certain demographic groups, particularly those less inclined to engage with digital tools. These limitations should be considered when interpreting the study’s results and their applicability to other contexts.

The findings of this study underscore the importance of digital teaching competencies in higher education, especially in the current digital age where virtual learning environments are increasingly common. The relationship between demographic factors and digital competencies highlights the need for targeted professional development initiatives. By understanding how variables such as age and academic profile affect digital competence, institutions can design more effective training programs that address the specific needs of educators. This, in turn, will lead to enhanced teaching practices and better student outcomes, as faculty are better equipped to integrate digital tools into their pedagogy.

### Recommendations

Based on the study’s findings, future research should focus on designing and implementing training programs that are specifically tailored to the digital teaching competence needs of university professors. These programs should emphasize both formative and procedural aspects, encouraging educators to actively produce and apply their learning in real-world contexts. The training should incorporate hands-on practice, allowing professors to develop digital competencies that meet the specific challenges of their teaching environments. Additionally, future studies should aim to employ random sampling techniques to create more representative samples, thus enhancing the validity and generalizability of the results. It is also recommended to explore the impact of digital competence on student learning outcomes, as this would provide further evidence of the importance of digital skills in education.

## Data Availability

Figshare: Data - Digital teaching competence of higher education professors.xlsx. https://doi.org/10.6084/m9.figshare.24084393.v3. ^70^ The project contains the following underlying data:
- Data- Digital teaching competence of higher education professors.xlsx Data are available under the terms of the Creative Commons Zero “No rights reserved” data waiver (CC0 1.0 Public domain dedication). Figshare: European Framework for Digital Competence of Teachers (DigComEdu) questionnaire. https://doi.org/10.6084/m9.figshare.24224065.v1. ^71^ The project contains the following extended data:
• QUESTIONNAIRE (DigComEdu).xlsx Data are available under the terms of the Creative Commons Attribution 4.0 International license (CC-BY 4.0).
