# Supplementary material for: Digital teaching competence of higher education professors: self-perception study in an Ecuadorian university
Source: F1000Res. 2024 Oct 29;12:1484. Originally published 2023 Nov 20. [Version 2] doi: 10.12688/f1000research.139064.2 (PMC11558163; doi:10.12688/f1000research.139064.2)
Supplement: Peer review history

# Reviewer report: Reviewer response for version 2

**DOI:** 10.5256/f1000research.172300.r336136

**Published:** 2024-11-12

**Authors:** Dirgha Raj Joshi

**Affiliations:** 1 Mahendra Ratna Campus Tahachal, Tribhuvan University,, Tribhuvan University, Kirtipur, Central Development Region, Nepal

Almost comments are addressed by the author hence I recommend it to index.

Is the work clearly and accurately presented and does it cite the current literature?

Yes

If applicable, is the statistical analysis and its interpretation appropriate?

Partly

Are all the source data underlying the results available to ensure full reproducibility?

Partly

Is the study design appropriate and is the work technically sound?

Yes

Are the conclusions drawn adequately supported by the results?

Yes

Are sufficient details of methods and analysis provided to allow replication by others?

Yes

Reviewer Expertise:

Educational technology, Applied Mathematics, Quantitative Research, SEM, Machine Learning

I confirm that I have read this submission and believe that I have an appropriate level of expertise to confirm that it is of an acceptable scientific standard.

# Reviewer report: Reviewer response for version 2

**DOI:** 10.5256/f1000research.172300.r336135

**Published:** 2024-11-06

**Authors:** Alexa Angelica Senior-Naveda

**Affiliations:** 1 Department of Humanities, Universidad de la Costa, Barranquilla, Colombia

The corrections were made completely and satisfactorily; in view of this, the article in its final version is considered an excellent contribution to knowledge.

Is the work clearly and accurately presented and does it cite the current literature?

Partly

If applicable, is the statistical analysis and its interpretation appropriate?

Partly

Are all the source data underlying the results available to ensure full reproducibility?

Yes

Is the study design appropriate and is the work technically sound?

Partly

Are the conclusions drawn adequately supported by the results?

Yes

Are sufficient details of methods and analysis provided to allow replication by others?

Partly

Reviewer Expertise:

Educación, Gestión educativa, Calidad educativa, Desarrollo sostenible,  Capital Social, Gobernanza, Redes.

I confirm that I have read this submission and believe that I have an appropriate level of expertise to confirm that it is of an acceptable scientific standard.

# Reviewer report: Reviewer response for version 1

**DOI:** 10.5256/f1000research.152308.r224295

**Published:** 2024-09-12

**Authors:** Alexa Angelica Senior-Naveda

**Affiliations:** 1 Department of Humanities, Universidad de la Costa, Barranquilla, Colombia

As a final concept, the evaluator considers that the inconsistencies present between the objectives set out in the introduction and the objectives that appear inserted in the theoretical framework must be resolved. To resolve this disagreement, it is suggested to correct the objectives of the introduction and delete from the theoretical framework section the same.

 Regarding the theoretical framework, the need to cite scientific articles that constitute empirical references for the application of the objectives of the 2030 agenda for sustainable development is highlighted in terms of the search for equality and social improvement based on use of technology in the Ecuadorian educational context

 The reviewer considers it necessary to correct the procedures declared as probabilistic in the methods section because in the sample estimation and sampling section the researchers declare that they carried out non-probabilistic sampling, which makes the methodological component inconsistent.

 Regarding the statistical parametric Cronbach's Alpha, this is not a result on the behavior of the variables, but rather a statistical requirement of the validity and reliability of the measurement instrument, on the discriminatory power of the items and the degree in which which the variance of each of the items measures the discriminatory power with respect to each indicator, as well as the variance of the entire instrument, which provides a measure of the variation of the variables studied. Therefore, these calculations should be part of a subsection of the methods section.

 It is also necessary to theoretically and scientifically support the adaptation of the data collection instrument to the Ecuadorian or similar context, since Cabero-Almenara and Palacios-Rodríguez adapted it to the European context, specifically to the Spanish context, which has cultural, demographic, socio-educational and economic conditions different from those of Ecuador.

 Once the previous corrections have been carried out both in the objectives declared in the introduction, as well as the objections in the methods, data analysis and results section; The article can be considered to have a coherent statistical analysis.

 It is highlighted that the data is available.

 Conclusions are inferred from the results.

 The article presents an important topic in the socio-educational area and of great interest today.

Is the work clearly and accurately presented and does it cite the current literature?

Partly

If applicable, is the statistical analysis and its interpretation appropriate?

Partly

Are all the source data underlying the results available to ensure full reproducibility?

Yes

Is the study design appropriate and is the work technically sound?

Partly

Are the conclusions drawn adequately supported by the results?

Yes

Are sufficient details of methods and analysis provided to allow replication by others?

Partly

Reviewer Expertise:

Educación, Gestión educativa, Calidad educativa, Desarrollo sostenible,  Capital Social, Gobernanza, Redes.

I confirm that I have read this submission and believe that I have an appropriate level of expertise to confirm that it is of an acceptable scientific standard, however I have significant reservations, as outlined above.

# Reviewer report: Reviewer response for version 1

**DOI:** 10.5256/f1000research.152308.r246734

**Published:** 2024-03-29

**Authors:** Dirgha Raj Joshi

**Affiliations:** 1 Mahendra Ratna Campus Tahachal, Tribhuvan University,, Tribhuvan University, Kirtipur, Central Development Region, Nepal

Title: Digital teaching competence of higher education professors: self-perception study in an Ecuadorian university

**Abstract**:

 Results in the abstract is just like conclusion hence it must contain some statistical values of core findings.

 Keywords: can add one additional keyword

**Background/Introduction**

 The research question should be added in the background section. There several paragraphs with out reference in the background section hence such paragraphs should be modified or remove. The theoretical explanation is good however missing literature review section hence some additional empirical literature is needed to add in the background section. Why researcher used each case capital like as “COMMITMENT” in the manuscript.

**Methodology**

 Several unnecessary information is mentioned under methodology section it will be better to organize it in the following manners as

 Research design 1-2 sentence

 Population sample and sampling technique -3-5 sentences

 Research instrument 3-5 sentences

 Variable information and validity and reliability of the instrument- one paragraph

 Data collection and data cleaning process- one paragraph

 Data analysis technique- one paragraph.

 Sample size is appropriate based on population however how it was calculated not clear hence should mentioned with reference. There are some gaps like why researcher used chi-square test instead of regression analysis.

**Results**

 Researcher made two factors in Table 7 however there is no any further analysis based on these factors. The items were dived into six categories in Table 3-Table 5 however the data demands only two categories hence further analysis based on gender and other variables on two factors is needed.

**Discussion**

 Discussion is appropriately explained however additional discussion and results are needed based on the results of two factors (Table 7). 

**Conclusion**

 Limitation, implication, and recommendation should be added in separate section.

**Citation and references**

 Need to check as per authors guideline.

**Language**

 Language should be improved.

Is the work clearly and accurately presented and does it cite the current literature?

Yes

If applicable, is the statistical analysis and its interpretation appropriate?

Partly

Are all the source data underlying the results available to ensure full reproducibility?

Partly

Is the study design appropriate and is the work technically sound?

Yes

Are the conclusions drawn adequately supported by the results?

Yes

Are sufficient details of methods and analysis provided to allow replication by others?

Yes

Reviewer Expertise:

Educational technology, Applied Mathematics, Quantitative Research, SEM, Machine Learning

I confirm that I have read this submission and believe that I have an appropriate level of expertise to confirm that it is of an acceptable scientific standard, however I have significant reservations, as outlined above.
